# DNA‐Binding Properties of Non‐Intercalating Water‐Soluble Organometallic Ir(III) Luminophores

**DOI:** 10.1002/chem.202500290

**Published:** 2025-05-02

**Authors:** Ibrahim S. Alkhaibari, Peter N. Horton, Simon J. Coles, Niklaas J. Buurma, Simon J. A. Pope

**Affiliations:** ^1^ School of Chemistry, Main Building Cardiff University Cardiff CF10 3AT UK; ^2^ Department of Chemistry, College of Science Qassim University Buraydah 52571 Saudi Arabia; ^3^ UK National Crystallographic Service, Chemistry University of Southampton Highfield Southampton SO17 1BJ UK

**Keywords:** calorimetry, DNA binding, docking, iridium complex, luminescence

## Abstract

A series of Ir(III) complexes, [Ir(C^N)_2_(en)]^+^ (where C^N = 2‐phenyl‐benzo[*d*]thiazolyl cyclometalating ligand; en = ethylene diamine), is reported with structural variation via a substituent (H, Me, OMe, Cl, OCF_3_) at the coordinated phenyl ring. The complexes were soluble in aqueous buffer, with solubility limits correlating inversely with the predicted logP. The complexes display efficient visible absorption at 400–500 nm (ε ∼ 5000 M^−1^ cm^−1^) due to charge‐transfer transitions and are triplet emitters in aerated buffer (*λ*_em_ = 529–540 nm; lifetimes up to 0.763 µs; *Φ*_em_ ≤ 12%). Each complex was investigated, via computational and biophysical experiments, in the context of DNA binding. According to UV‐visible titrations, the cationic complexes bind to DNA with apparent affinities ranging from 6 × 10^4^ to 5 × 10^5^ M^−1^ with apparent binding site sizes between 0.4 and 1.0 base pairs. Isothermal titration calorimetry (ITC) showed that complexes [Ir(**L1‐3**)_2_(en)]Cl bind to DNA in two types of binding sites, viz., a high‐affinity (10^7^–10^8^ M^−1^) binding site with characteristics of minor or major groove binding and a low‐affinity binding site (10^5^ M^−1^) with characteristics of non‐specific binding to negatively charged DNA, with binding supported by hydrophobic interactions between complexes.

## Graphical abstract

A series of water‐soluble, organometallic iridium(III) complexes has been studied with respect to their DNA binding behavior. A combined biophysical and computational study reveals the subtle interplay of non‐intercalative DNA binding events that are modulated by small changes in ligand structure.

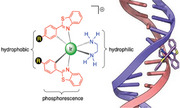

## 1 Introduction

The biological applications of phosphorescent organometallic complexes^[^ ^1^ ^]^ have been well established over the last 20 years or so, overcoming perceptions about incompatible solubility characteristics and toxicity, as well as complex instability in the presence of air and water.^[^ ^2^ ^]^ In particular, cell‐imaging research that uses confocal fluorescence microscopy^[^ ^3^ ^]^ has been well established for emissive organometallics, including Ir(III) systems,^[^ ^4^ ^]^ and exploits their advantageous tunable luminescence character.^[^ ^5^ ^]^ The advantages offered by Ir(III) complexes^[^ ^6^ ^]^ include the ease of tuning or adapting the ligands, which can be added in a stepwise manner, to control important physical properties such as solubility and charge, as well as the inherent photophysical attributes^[^ ^7^ ^]^ that can allow luminescence wavelengths in the deep red region.^[^ ^8^ ^]^ These amenable design features, married with kinetic inertness and high photostability, have positioned luminescent Ir(III) complexes as viable options within the bioimaging tool kit.^[^ ^9^ ^]^ Despite their relatively common deployment in cellular studies and bioimaging, the fundamental way in which emissive Ir(III) complexes interact with common biomolecules, such as DNA, is far less well‐studied.^[^ ^10^ ^]^ This becomes especially relevant when the intracellular targets are the nucleus or mitochondria,^[^ ^11^ ^]^ both of which contain DNA, and when one considers the phototoxicity of Ir(III) species.^[^ ^12^ ^]^

Luminescent Ru(II) polypyridine complexes,^[^ ^13^ ^]^ and in particular the “light switch” behavior of dppz (dipyridophenazine) derivatives,^[^ ^14^ ^]^ are perhaps the archetypal metal complexes for studying DNA binding^[^ ^15^ ^]^ and evolutions of the design have been extensively developed.^[^ ^16^ ^]^ Remarkably, the comparable behaviors of closely related Ir(III) systems are far less reported. Thomas and co‐workers have studied isostructural Ir(III) analogs of the famous Ru(II) dppz systems, including a cyclometalated version of dppz, and shown that they bind to DNA with affinities that are comparable to the classical Ru(II) (dppz) benchmarks.^[^ ^17^ ^]^ However, as noted,^[^ ^18^ ^]^ heteroleptic bis‐cyclometalated Ir(III) complexes, [Ir(C^N)_2_(N^N)]^+^, typically possess a lower cationic charge which, even as their chloride salts, can restrict solubility in aqueous buffer media.

**Figure 1:**
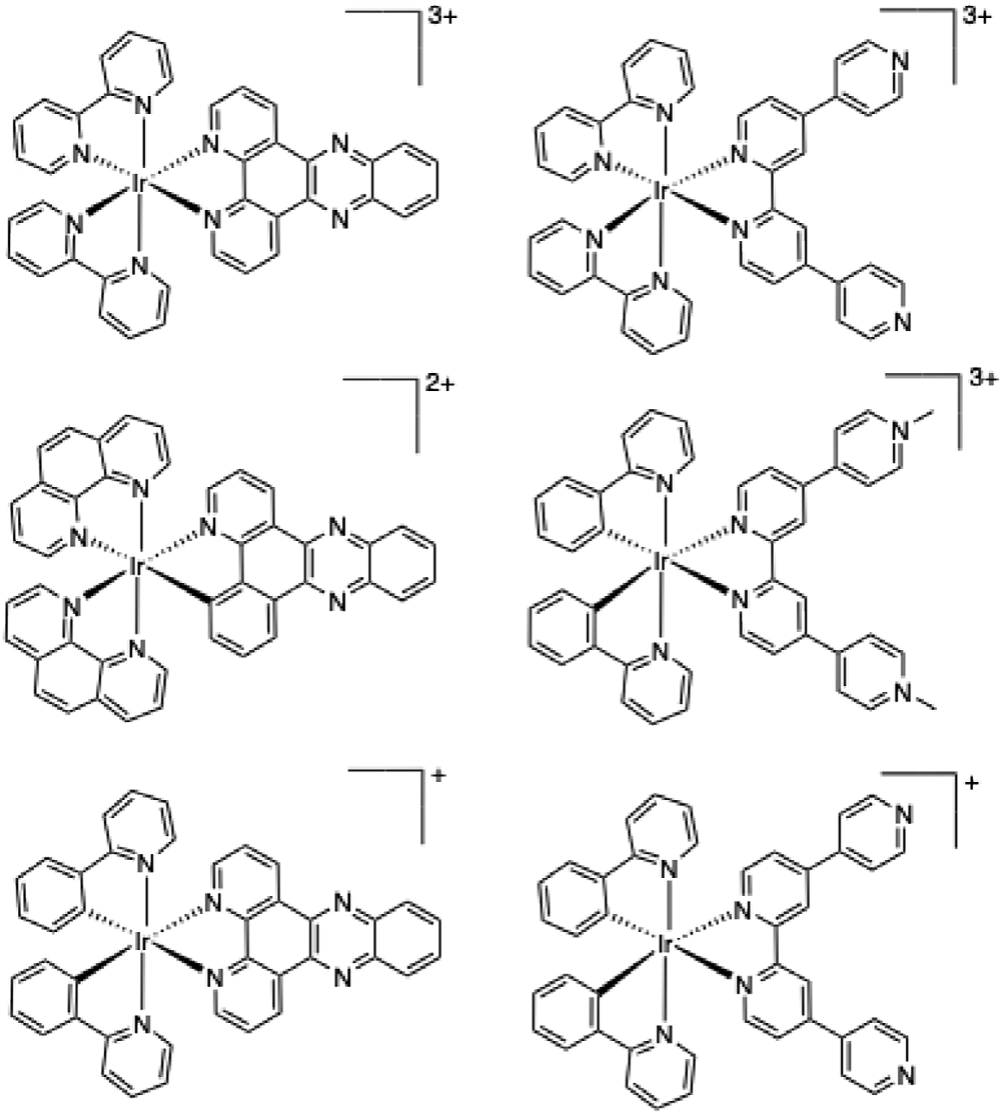
Previous examples of cationic Ir(III) complexes used in DNA binding studies, including dppz variants (left).

Low concentrations of complexes in solutions potentially limit the choice of techniques available for experiments such as DNA‐binding studies. For example, isothermal titration calorimetry typically requires high complex concentrations of at least the order of 100 µM, and UV‐visible titrations typically require concentrations of the order of 10 µM. Whereas stock solutions of complexes in DMSO can be made, this then introduces DMSO in the experiments of interest. Moreover, aqueous solutions prepared by diluting a concentrated stock solution in DMSO may be unstable and result in unexpected precipitation. Good aqueous solubility therefore provides benefits in terms of solution stability and compatibility with a variety of techniques. In the case of cell imaging studies, however, there is a trade‐off. Numerous studies have shown that a lower cationic charge is beneficial for cellular imaging studies^[^ ^3^ ^]^ that are implicitly reliant upon efficient cellular uptake. Nevertheless, compounds with higher cationic charges have still achieved success in cellular imaging including showing charge‐dependent targeting.^[^ ^19^ ^]^ Therefore alternative polypyridine variants, e.g. [Ir(N^N)_3_]^3+^, which carry a higher cationic charge and thus are analogous to the Ru(II) complexes discussed earlier, have attracted attention. General strategies to promote the aqueous solubility of Ir(III) organometallics are known,^[^ ^20^ ^]^ but for prospective Ir(III) DNA binders the use of charged ligands, or ligands that are protonated under physiological conditions, (relevant structures shown in Figure 1) are attractive.^[^ ^21^ ^]^

Highly planar aromatic ligands (such as dppz or phen derivatives^[^ ^22^ ^]^) commonly promote DNA binding through an intercalation mode. However, other binding modes have been reported for Ir(III) complexes that employ 2,2′:4,4″:4′,4′′′‐quaterpyridyl ligands (Figure 1) including both intercalation and groove binding.^[^ ^17^ ^]^ One disadvantage of the polypyridine complexes of Ir(III) is that [Ir(N^N)_3_]^3+^ species often lack the advantageous (certainly in a biological sense) photoluminescent properties of their cyclometalated relatives.^[^ ^23^ ^]^

In this context, we wished to investigate the DNA‐binding behavior of cationic Ir(III) complexes that marry aqueous solubility and favorable emissive characteristics. We were inspired by the pioneering work of Lo and co‐workers in their development of chelated diamine derivatives^[^ ^24^ ^]^ of phosphorescent Ir(III) complexes that were subsequently used for cellular bio‐imaging.^[^ ^25^ ^]^ Thus, by combining polyaromatic cyclometalating ligands, a hydrophilic ethylene diamine ancillary ligand, and a chloride counter ion (i.e., [Ir(C^N)_2_(en)]Cl, where en = ethylene diamine) we show that sufficient aqueous solubilities were achieved to enable biophysical studies with DNA. In addition, the complex design allowed a point of structural variation via substitution of the cyclometalating ligand, providing an opportunity to fine‐tune the spectroscopic and the amphiphilic nature of the complex. The complexes described herein were investigated for their DNA‐binding behavior using a range of biophysical techniques, including isothermal calorimetry (ITC) and computational docking studies.

## 2 Results and Discussion

### 2.1 Synthesis of the Complexes

**Scheme 1:**
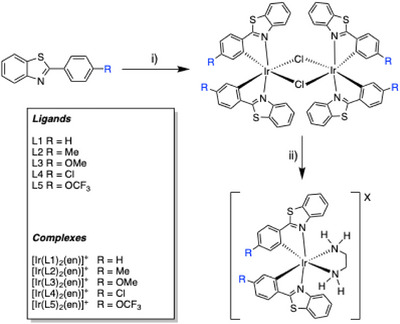
General synthetic scheme for the complexes. Reagents and conditions: i) 0.5 eq. IrCl₃·xH₂O, 2‐ethoxyethanol, water, heat to reflux; ii) excess ethylenediamine, 2‐ethoxyethanol, heat to reflux.

The heteroleptic complexes in this study possess a hydrophobic, conjugated 2‐phenyl‐benzo[*d*]thiazolyl cyclometalating ligand architecture and a hydrophilic ethylene diamine ancillary ligand. The target complexes are cationic and thus also amenable to a choice of counter ion. As noted earlier, while the ethylene diamine ancillary ligand can impart water solubility, the photoluminescence properties of the complexes are typically determined by the nature of the cyclometalating ligand; in this case substituted 2‐phenyl‐benzo[*d*]thiazole ligands were selected. The overall synthetic pathway to the complexes is shown in Scheme 1. The 2‐phenylbenzo[*d*]thiazole species have been previously reported,^[^ ^26^ ^]^ but are repurposed^[^ ^27^ ^]^ here as cyclometalating ligands (**L1‐L5**). The Ir(III) complexes were synthesized using the approach described previously^[^ ^28^ ^]^ to yield, first, the μ‐dichloro bridged dimer species, [(**L**)_2_Ir(μ‐Cl)}_2_]. Subsequent addition of excess ethylene diamine in 2‐ethoxyethanol followed by anion exchange using aqueous NH_4_PF_6_ yielded the crude monometallic cationic complexes, [Ir(**L**)_2_(en)]PF_6_, which were further purified using recrystallization to give the five complexes as colored, air‐stable solids.

The proposed formulae of the isolated hexafluorophosphate salts of the complexes were investigated using a range of spectroscopic and analytical techniques. First, ^1^H NMR spectroscopy showed that the two C^N ligands are equivalent and present one set of aromatic ^1^H resonances which were generally quite well resolved. In all cases, the influence of cyclometalation was evidenced by a relatively upfield signal (typically between 6.0 and 6.5 ppm) assigned to the hydrogen position adjacent to the site of metalation. In all cases there were two distinct sets of N*H* resonances (each integrating to 2H) at approximately 4.5 and 5.5 ppm, respectively, representing a significant downfield shift upon coordination to Ir(III), consistent with previous reports.^[^ ^29^ ^]^ The conformation of the coordinated en ligand also results in two distinct C*H* signals between 2 and 3 ppm. ^13^C{^1^H} NMR data were also obtained for each complex and revealed a furthest downfield resonance ca. 182 ppm, assigned to the benzothiazolyl carbon. The ethylene diamine carbons appeared as a single resonance ca. 45 ppm. The anticipated C–F coupling arising from the trifluoromethane group in [Ir(**L5**)_2_(en)]PF_6_ was difficult to fully resolve due to overlapping aromatic resonances, but evidence for both ^1^ *J*_CF_ and ^2^ *J*_CF_ were observed at 115–130 ppm. All relevant NMR spectra are available in the SI (Figures S1–S10). The ^19^F{^1^H} NMR spectrum of [Ir(**L5**)_2_(en)]PF_6_ also revealed a singlet at −56.27 ppm, which was consistent with an aromatic CF_3_ species,^[^ ^30^ ^]^ and very close to the value of the free ligand, **L5** (−56.67 ppm); a doublet was also observed at −70.14 ppm (^1^ *J*_FP_ ∼ 755 Hz) correlating with the hexafluorophosphate anion. The Ir(III) complexes gave excellent HRMS data (Figure S11) with *m/z* values that were consistent with the cationic complex fragment, [Ir(**L**)_2_(en)]^+^ in each case, as well as evidence for the loss of ethylene diamine. Satisfactory elemental analyses were obtained for each of the complexes, and supporting IR spectra (Figure S12, SI) were recorded that confirmed the presence of the key functional groups within the ligands (for example, typically two N‐H stretches at 3200–3400 cm^−1^) and the hexafluorophosphate counter ion (ca. 830 cm^−1^).

### 2.2 X‐ray Crystal Structures of [Ir(L1)_2_(en)]PF_6_, [Ir(L2)_2_(en)]PF_6_ and [Ir(L4)_2_(en)]PF_6_

**Figure 2:**
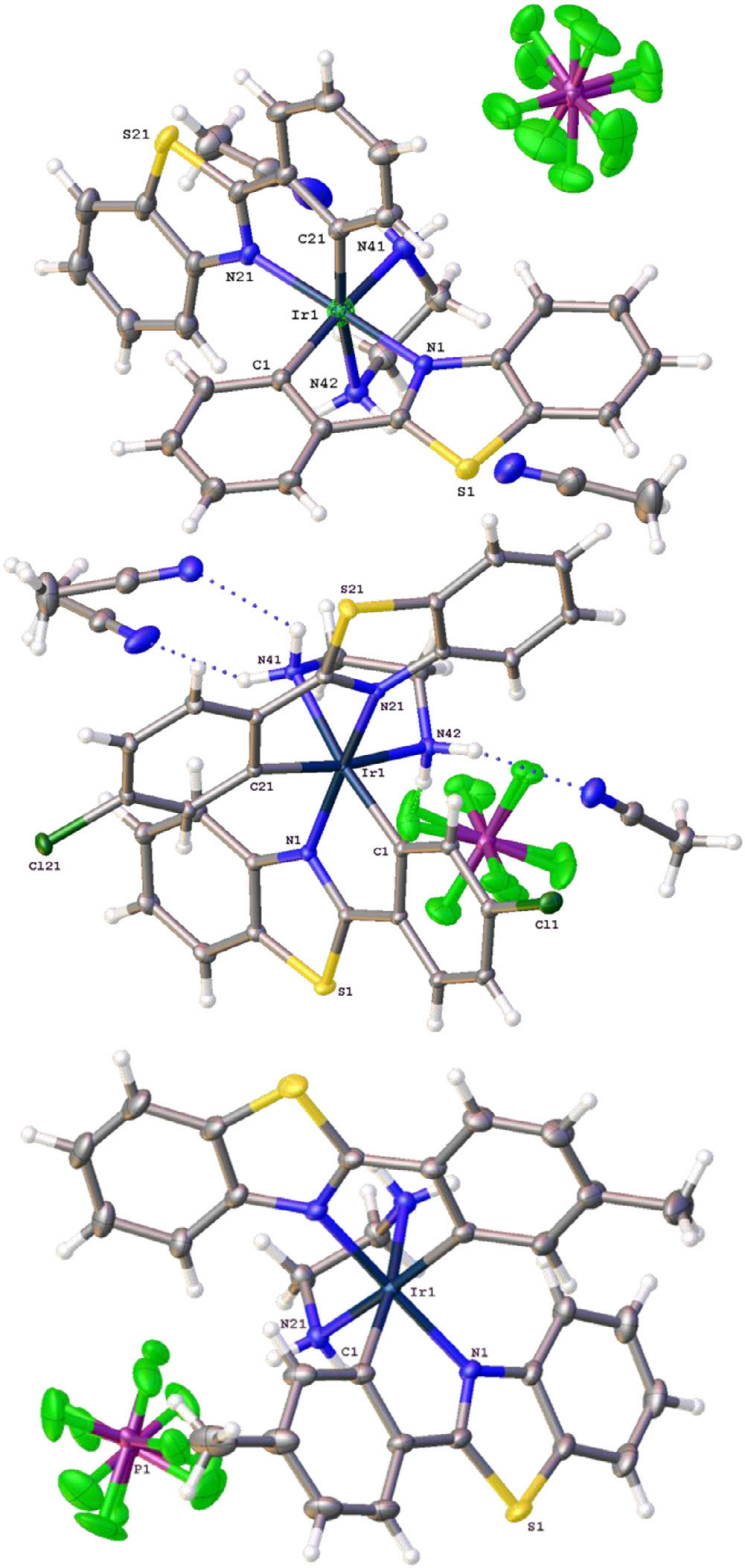
Structures obtained from the single crystal X‐ray diffraction studies of (top‐to‐bottom) [Ir(**L1**)_2_(en)]PF_6_, [Ir(**L2**)_2_(en)]PF_6_ and [Ir(**L4**)_2_(en)]PF_6_ Thermal ellipsoids drawn at 50%.

**Figure 3:**
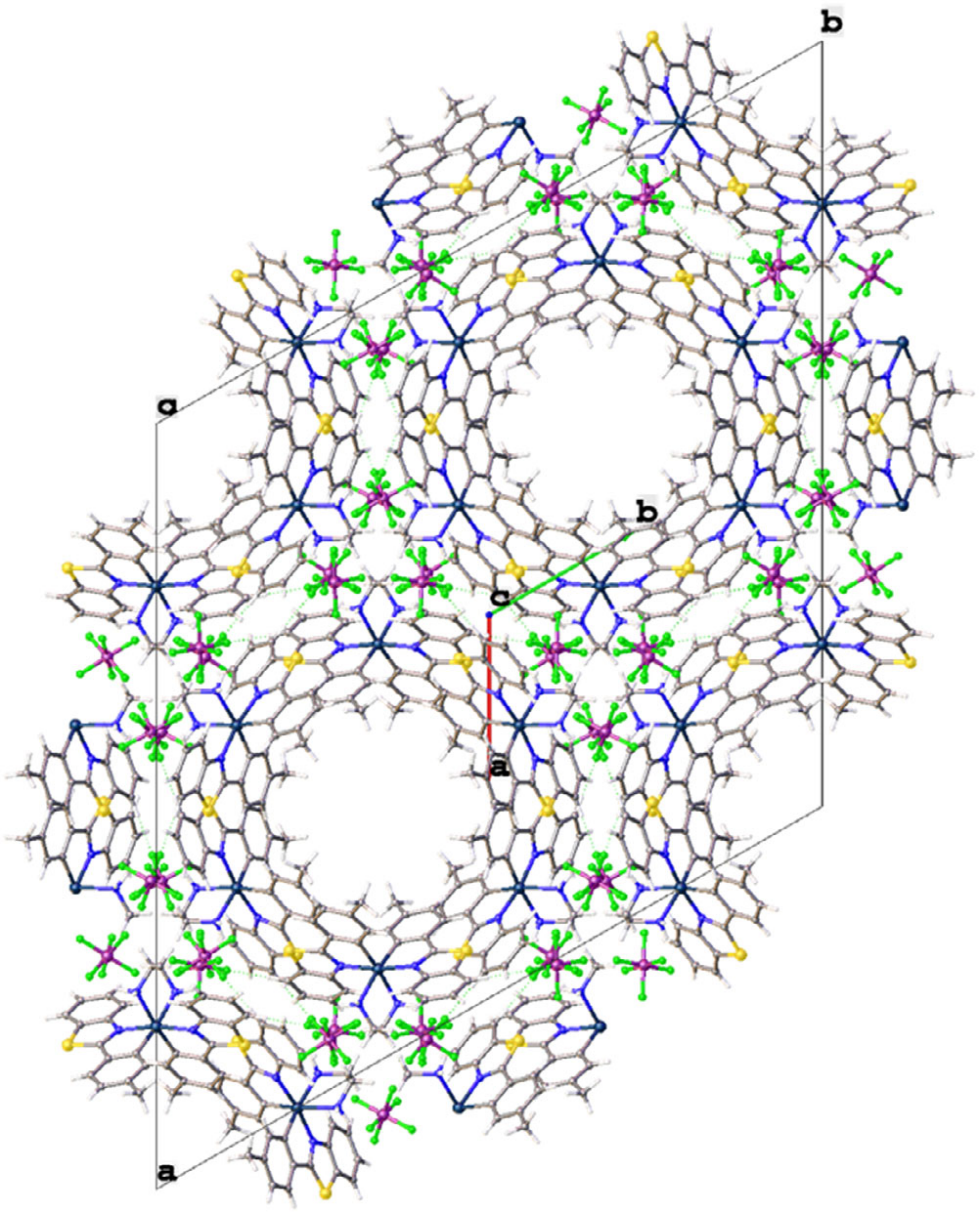
Packing diagram of [Ir(**L2**)_2_(en)]PF_6_ viewed along the *c* axis.

Suitable crystals (typically orange in appearance) were obtained for X‐ray diffraction studies on three of the complexes using vapor diffusion of ^i^Pr_2_O into concentrated MeCN solutions. The data collection parameters are shown in Table S1. The structure solutions of the diffraction data confirmed the proposed formulations and geometries for the complexes (Figure 2). All three structures contain solvent; for [Ir(**L1**)_2_(en)]PF_6_ and [Ir(**L4**)_2_(en)]PF_6_, the MeCN were well defined and readily identified, but for [Ir(**L2**)_2_(en)]PF_6_, they could not be located and solvent masking was used. The 2‐phenyl‐benzo[*d*]thiazole cyclometalating ligands adopt a mutually *cis*‐C,C‐arrangement to the Ir–C bonds. The five‐membered chelate rings thus impart a slightly distorted octahedral geometry at iridium. The six coordination sphere bond lengths (Table 1; for bond angles see Table S2) are comparable with a number of previous reports on related complexes,^[^ ^31^ ^]^ as well as earlier work on ethylene diamine Ir(III) species.^[^ ^32^ ^]^ Each of the structures shows that the Ir‐N_en_ bond lengths are typically longer (ca. 2.19 Å) than the corresponding coordinate bonds to the benzothiazole ligand (typically around 2.07 Å). The Ir–C bond lengths are the shortest within the coordination sphere for these complexes. For [Ir(**L4**)_2_(en)]PF_6_, there are a number H‐bonding interactions that involve the coordinated ethylene diamine, PF_6_ counter anion and solvent of recrystallization (contacts described by N41–N51 = 3.0882(17) Å; N41–N61 = 3.1553(19) Å; N42–F83 = 3.031(9) Å; N42–N71 = 3.1031(17) Å; N42–F83A = 3.000(14) Å). Interestingly, the packing diagram for [Ir(**L2**)_2_(en)]PF_6_ revealed an aesthetically pleasing arrangement where neighboring cationic complex units are organized in a way that demonstrate cylindrical ordering when viewed along the *c* axis (Figure 3); the channels are filled with highly disordered solvent acetonitrile.

**Table 1:** The coordination sphere bond lengths (Å) for the three structures.

[Ir(**L1**)_2_(en)]PF_6_	Ir1‐N1	2.0664(8)	Ir1‐N42	2.1928(10)
	Ir1‐N21	2.0793(8)	Ir1‐C1	2.0082(10)
	Ir1‐N41	2.1892(9)	Ir1‐C21	2.0085(10)
[Ir(**L2**)_2_(en)]PF_6_	Ir1‐N1’	2.0652(9)	Ir1‐N21	2.1933(10)
	Ir1‐N1	2.0652(9)	Ir1‐C1’	2.0100(12)
	Ir1‐N21’	2.1932(10)	Ir1‐C1	2.0102(12)
[Ir(**L4**)_2_(en)]PF_6_	Ir1‐N1	2.0726(9)	Ir1‐N42	2.1789(10)
	Ir1‐N21	2.0685(9)	Ir1‐C1	2.0052(11)
	Ir1‐N41	2.1927(10)	Ir1‐C21	2.0160(11)

**Table 2:** Absorption and emission data of the Ir(III) complexes.^[^ ^a^ ^]^

	σ_p_^[^ ^33^ ^]^	Absorbance λ_max_ [nm]	Emission λ [nm]^[^ ^b^ ^]^	Lifetime [µs]^[^ ^c^ ^]^	Quantum yield [%]^[^ ^d^ ^]^
[Ir(**L3**)_2_(en)]Cl	−0.27	328, 374, 422	529	0.763	9.5
[Ir(**L2**)_2_(en)]Cl	−0.17	326, 378, 430	538	0.399	12.0
[Ir(**L1**)_2_(en)]Cl	0	324, 382, 432	540	0.563	7.8
[Ir(**L4**)_2_(en)]Cl	0.22	326, 380, 420	536	0.275	6.6
[Ir(**L5**)_2_(en)]Cl	0.54	322, 382, 424	537	0.588	5.3

^[a]^ All measurements obtained in aerated MOPS buffer (25 mM MOPS, 50 mM NaCl, pH 7.00) at 25 °C;

^[b]^ λ_ex_ = 410 nm;

^[c]^ observed photoluminescence lifetime, λ_ex_ = 295 nm;

^[d]^ [Ru(bipy)_3_][PF_6_]_2_ serving as the reference in aerated MeCN, and the quantum yield (Φ) is 1.8%.

Having successfully isolated and characterized the cationic complexes as hexafluorophosphate salts, counter‐ion exchange was performed to enable the subsequent biophysical studies with DNA. Each of the complexes was converted to its chloride salt using amberlite IRA‐402 Cl‐form resin; the ion exchange process was monitored using ^19^F NMR spectroscopy and confirmed via elemental analysis.

### 2.3 Electronic Properties of the Complexes

**Figure 4:**
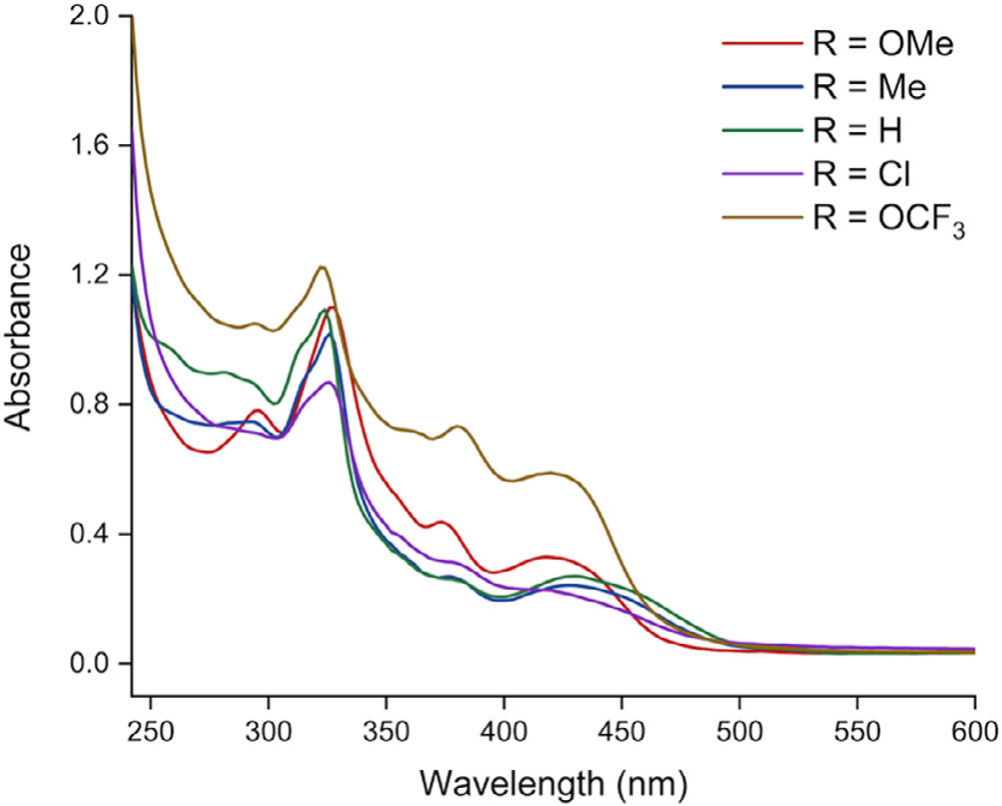
UV‐vis absorption spectra of the Ir(III) complexes in aerated MOPS buffer (25 mM MOPS, 50 mM NaCl, pH 7.00) at 25 °C.

The UV‐vis absorption data for aerated solutions of [Ir(**L**)_2_(en)]Cl dissolved in buffer (25 mM MOPS and 50 mM NaCl at pH 7.00) were recorded (see Experimental section for details). The data are presented in Table 2 and Figure 4. The spectra generally show that the complexes absorb strongly < 450 nm. Within the UV region, there are two main peaks at ca. 325 and 375 nm which vary in relative absorbance; in some cases, the latter band is less distinct and appears as a shoulder on the lower energy tail of the first absorption band. These features are primarily attributed to spin‐allowed ligand‐centered transitions associated with the conjugated, aromatic ligands and are π→π* in nature. The subtle variance in the appearance of the ligand bands is likely due to the different substituents present on the phenyl ring. A visible absorption band was also noted with a peak position between 420 and 435 nm (molar absorption values, determined in triplicate, are given in Table S4); again, the relative absorbance and broadness of the band vary across the series of complexes, which accounts for the slight differences in color observed for the complexes. This band was attributed to a spin‐allowed (see SI for molar absorption determinations) metal‐to‐ligand charge transfer transition(s) (^1^MLCT) which must encompass some Ir_5d_→L_bt_ character (where bt = benzothiazole). Within the broad absorption envelope, a significant low energy tail (ε < 1000 M^−1^ cm^−1^) was noted, especially for [Ir(**L2**)_2_(en)]PF_6_, which is probably due to a spin‐forbidden S_0_ → T_1_ contribution mediated by the heavy Ir atom.^[^ ^34^ ^]^ Across the series of complexes the absorption spectra are broadly comparable suggesting the type of substituent present in the cyclometalated ligand produces a minor perturbation of the absorption character of the complexes. The absorption features of these complexes compare with [Ir(ppy)_2_(bipy)]^+^ (where ppy = 2‐phenylpyridine),^[^ ^7a^ ^]^ but with more efficient molar absorption in the 350–400 nm range, which is likely due to the conjugation within the 2‐phenyl‐benzothiazole cyclometalating ligands.

**Figure 5:**
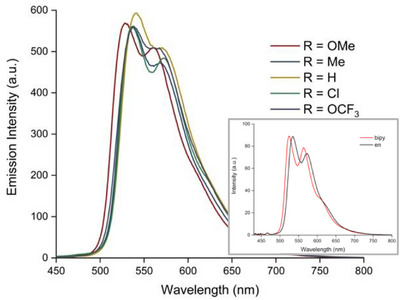
Main: photoluminescence spectra (λ_ex_ = 410 nm) of the complexes in aerated MOPS buffer (25 mM MOPS, 50 mM NaCl, pH 7.00) at 25 °C. Inset: A comparison of the emission spectra of [Ir(**L4**)_2_(N^N)]PF_6_ where N^N = en or bipy (MeCN, λ_ex_ = 410 nm) is shown inset.

The photoluminescence properties of the complexes were recorded using aerated MOPS buffer solutions described above. First, each of the complexes was emissive in the visible region between 529 and 540 nm; these complexes are green emitters. Figure 5 shows that the appearance of the spectra is comparable with a structured emission band comprising two peak features. The spectral profile of these complexes suggests that they may possess some ligand‐centered triplet character; previous studies on related benzothiazole complexes of Ir(III) have proposed an admixture of ^3^IL and ^3^MLCT excited states.^[^ ^25^ ^]^ The observed lifetimes for the complexes vary in the range 0.275–0.763 µs, confirming that phosphorescent character is demonstrated under aqueous buffer conditions. The luminescence quantum yields (5.3–12.0%) are moderate values consistent with a phosphorescent species measured under ambient conditions. The influence of the ethylene diamine co‐ligand was demonstrated by comparing the emission spectra for two complexes, viz. [Ir(**L4**)_2_(en)]PF_6_ versus [Ir(**L4**)_2_(bipy)]PF_6_, in MeCN (see inset, Figure 5). It is noteworthy that the emission maximum for [Ir(**L4**)_2_(en)]PF_6_ is slightly bathochromically shifted relative to [Ir(**L4**)_2_(bipy)]PF_6_ although the peak shape and vibronic features are closely replicated. The shift can be attributed to the influence of the ancillary ligand (in this case en vs bipy) upon the HOMO level of the complex which is likely to comprise significant Ir 5d orbital character. Critically, the comparison of the complexes shows that the cyclometalating ligands are crucial in defining the emission properties and that variation of the ancillary ligand is possible without loss of favorable photophysical properties. To place these observations in context, [Ir(ppy)_2_(bipy)]^+^ emits with a broad featureless peak at 602 nm that is assigned to an admixture of ^3^MLCT/^3^LLCT excited states.

### 2.4 Interactions With DNA: Computational and Experimental Studies

**Figure 6:**
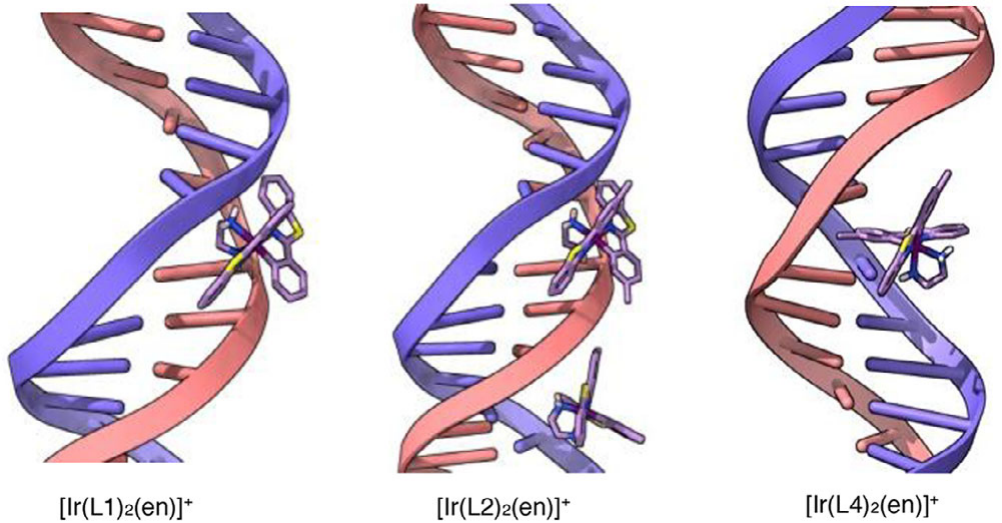
Complexes were docked with the d(ATCGAGACGTCTCGAT)₂ structure with a preformed intercalation gap. Note both major and minor groove binding modes are predicted for [Ir(**L2**)_2_(en)]^+^.

The physical properties of the complexes, including UV‐vis absorption spectra and long‐lived phosphorescence, mean that these compounds may have applications in bioimaging and/or biosensing. We therefore continued with a combined computational and experimental biophysical analysis of the DNA binding characteristics of the complexes. Prior to an experimental biophysical study, a docking investigation (using AutoDockTools 1.5.4.^[^ ^35^ ^]^ and AutoDock Vina^[^ ^36^ ^]^) was undertaken on the three complexes for which structural X‐ray data were obtained, to explore their potential interactions with DNA. Docking studies used a duplex DNA structure d(ATCGAGACGTCTCGAT)₂ with a pre‐formed intercalation gap^[^ ^37^ ^]^ as the target. The docking models for the selected complex structures were derived from the CIF files obtained from the X‐ray crystal structures of [Ir(**L1**)_2_(en)]^+^, [Ir(**L2**)_2_(en)]^+^ and [Ir(**L4**)_2_(en)]^+^. However, as Ir has not been suitably parameterized, it was replaced with Co to generate structures compatible with the parameterization in AutoDock Vina. Importantly, the coordination environment around the metal ion is kept rigid during docking so replacing Ir with Co does not affect the shape of the complex. Up to three binding modes with the lowest energy are presented for each complex, excluding those with the same binding position (Figure 6 and Table 3).

**Table 3:** The top three binding modes^[^ ^a^ ^]^ predicted using molecular docking.

Complex	Interaction type(s)	Affinity [kcal mol^−1^]^[^ ^b^ ^]^
[Ir(L1)_2_(en)]^+^	all minor groove	−7.9, −7.7, −7.7
[Ir(L2)_2_(en)]^+^	both minor and major groove	−7.8, −7.8, −7.7
[Ir(L4)_2_(en)]^+^	all major groove	−7.7, −7.7,−7.6

^[a]^ Ranking of the binding modes produced by Vina;

^[b]^ binding affinities are reported to 0.1 kcal mol^−1^ by Vina.

The calculations suggest that each of the complexes is a groove binder, with the possibility that the nature of the substituent on the cyclometalating ligand can influence a preference for major or minor grooves of the DNA. Although there appears to be a preference for binding close to the preformed intercalation gap, the results from the docking studies suggest that intercalation‐style interactions do not make a major contribution to the affinity of these complexes for DNA. The predicted absence of intercalative binding is attributed to the structures of the complexes which, as a result of the complexation geometries of the ligands around the metal center, lack suitably exposed flat aromatic moieties to slide in between the base pairs; in other words, the complex geometries don't allow sufficient stacking interactions between the ligand and the base pairs around the intercalation gap. Similar non‐intercalative minor‐groove binding modes have been observed in a crystal structure for Δ‐[Ru(phen)_2_(dppz)]^2+^ bound to DNA.^[^ ^38^ ^]^ We note that these crystal structures involved an extent of DNA structural deformation that docking studies do not reproduce. The effect of the coordination geometry on the DNA‐binding mode was further probed using the free 2‐phenyl‐benzothiazole ligand (**L1**) which, again, was docked independently using AutoDock Vina against a DNA structure with a pre‐formed intercalation gap. The results predict that the interaction between **L1** and DNA occurs through intercalation (Figure S13) which is clearly different from the corresponding complex.

We next estimated the solubility limits of the complexes in buffer (25 mM MOPS, pH 7.0, 50 mM NaCl) from saturated solutions, using the molar absorption coefficients in Table S4 (see also Figures S14–18). Under these conditions, the solubility limits for the complexes were found to be 978, 327, 739, 100, and 75 µM, respectively. These values mirror the trend predicted (via logP^[^ ^39^ ^]^) for the relative hydrophobicities of the cyclometalating ligands, which is ultimately controlled by the ligand substituent (H vs Me vs OMe vs Cl vs OCF_3_).

**Table 4:** Apparent DNA‐binding affinities of the iridium complexes, [Ir(**L**)_2_(en)]Cl, in buffer (25 mM MOPS, 50 mM NaCl, pH 7.0) at 25 °C.

	L1	L2	L3	L4	L5
*K* / 10^5^ M^−1^	4.3 ± 5.3	4.2 ± 3.7	4.6 ± 2.3	0.8 ± 0.7	0.6 ± 0.3
Δ*ε* / 10^3^ M^−1^ cm^−1^	−2.7 ± 0.2	−3.2 ± 0.1	−5.9 ± 0.2	−3.6 ± 0.2	−6.6 ± 0.2
*Ε* ^[^ ^a^ ^]^ / 10^4^ M^−1^ cm^−1^	2.44	2.876	3.452	2.874	3.245
binding site size / base pairs	0.70 ± 0.15	0.64 ± 0.05	0.76 ± 0.04	0.43 ± 0.19	0.92 ± 0.18

^[a]^ Fitted error margins on *ε* are not reported because *ε* was used to determine the complex concentration in solution from observed absorbance of the complex solution in the absence of DNA.

**Figure 7:**
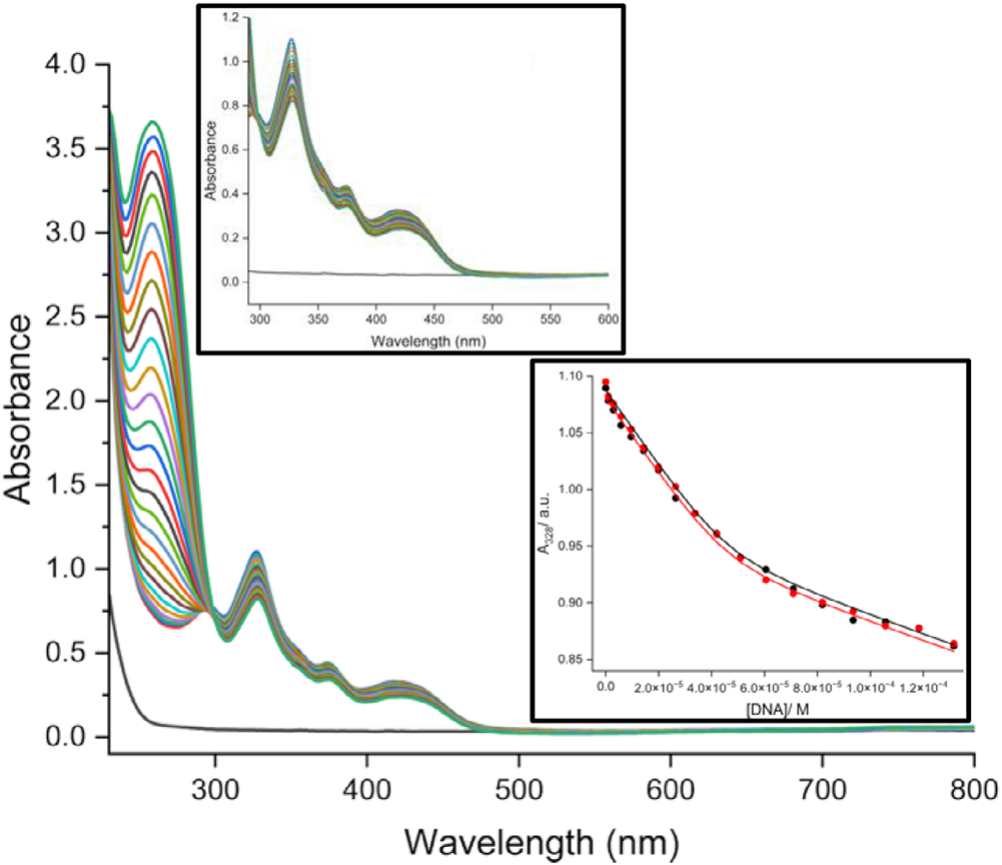
Example of a UV‐vis titration for [Ir(**L3**)_2_(en)]Cl (31.52 µM) with sequential aliquots of FSDNA in buffer (25 mM MOPS, 50 mM NaCl, pH 7.0) at 25 °C. Insets show an expansion (top left) of the complex absorption bands and the absorbance data at 328 nm fitted globally in terms of the multiple independent binding sites model.

We then studied the interactions of the complexes with double‐stranded DNA using UV‐visible titrations. Briefly, solutions of 44.14, 31.52, 35.17, 37.55, and 29.62 µM for the five complexes [Ir(**L1‐5**)_2_(en)]Cl, respectively, were titrated (in duplicate) with fish sperm DNA (FSDNA) at 25 °C (Figure 7 and Figures S19–22). The interactions between these complexes and DNA turned out not to involve a single type of binding site (vide infra). Nevertheless, the data from the UV‐visible titrations were provisionally analyzed globally using the multiple independent binding sites (MIS) model to determine apparent binding affinities and apparent binding site sizes (Table 4).

The apparent binding parameters (Table 4) are apparent affinities for DNA ranging from 6 × 10^4^ to 4.6 × 10^5^ M^−1^ with apparent binding site sizes ranging between 0.4 and 1.0 base pairs. The measured affinities confirm the binding of the complexes with DNA, but the stoichiometries suggest binding sites that correspond to the interaction of up to one iridium complex per DNA base, i.e., binding until charge cancellation occurs between the DNA and DNA‐bound complexes. In our experience,^[^ ^40^ ^]^ DNA‐binding compounds and complexes that display this behavior in UV‐visible titrations often involve multiple binding sites. The apparent stoichiometry corresponding to full charge cancellation then often reflects a secondary non‐specific binding event following a higher‐affinity more specific binding mode. Considering that the UV‐visible titration data are reproduced well by a model involving a minimum number of parameters, we decided against analysis using a model encompassing additional optimizable parameters because such a model would result in significant parameter correlation and therefore meaningless values for the optimized parameters.

**Figure 8:**
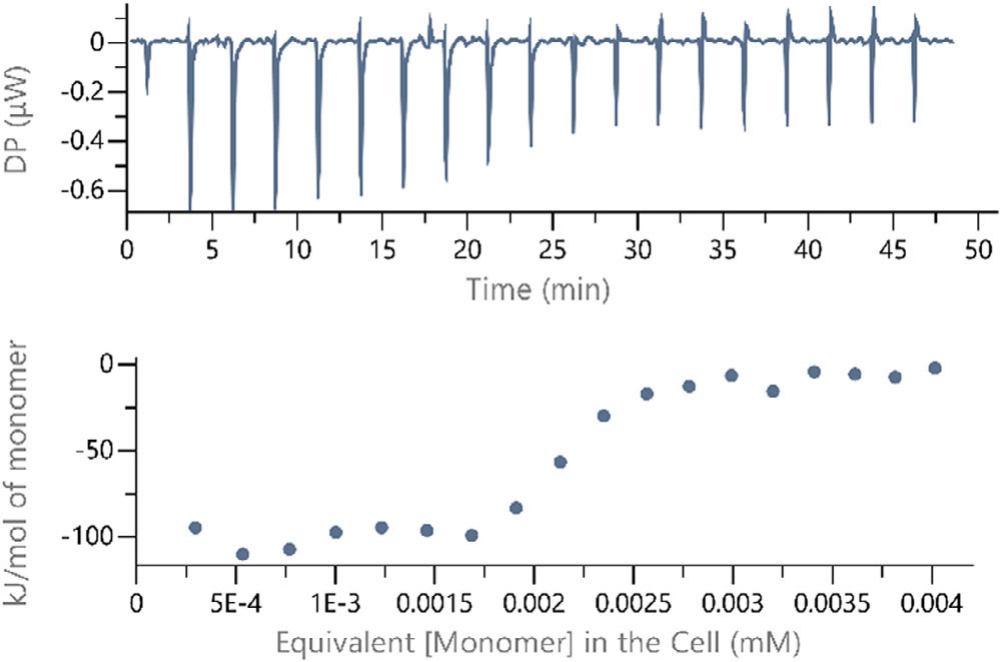
Raw and integrated heat effects for dilution of a 25 µM solution of [Ir(**L1**)_2_(en)]Cl in buffer (25 mM MOPS, 50 mM NaCl, pH 7.0) into matched buffer at 25 °C.

To explore whether multiple binding modes are indeed present for these complexes interacting with DNA, we turned to isothermal titration calorimetry (ITC). ITC typically involves titrating a relatively concentrated solution of the binder into a more dilute solution of DNA (instead of titrating a relatively concentrated DNA solution into a more dilute solution of the binder). Therefore, the possible self‐aggregation of the DNA binders was explored through dilution experiments in which solutions of a complex at representative concentrations in buffer (25 mM MOPS, pH 7.0, 50 mM NaCl) were diluted into matched buffer in the calorimeter cell. Only three of the complexes, [Ir(**L1‐3**)_2_(en)]Cl demonstrated sufficient solubilities to be compatible with this biophysical study. The ITC dilution curve for [Ir(**L1**)_2_(en)]Cl is shown in Figure 8.

Figure 8 shows that the dilution heat effects vary sigmoidally around a concentration of 2.1 µM. Further experiments at different complex concentrations confirm this sigmoidal behavior of the dilution heat effects around a critical concentration. The critical aggregation concentration (CAC) of 2.1 µM is remarkably low but, as suggested by us^[^ ^41^ ^]^ and others^[^ ^42^ ^]^ before, we note that aggregation at low concentrations is likely to be missed in studies and micromolar CACs are therefore under‐reported in the literature. Nevertheless, self‐assembled nanoparticles of organic fluorophores with micromolar CACs have been reported.^[^ ^43^ ^]^

CACs are typical for cooperative aggregation processes, such as micellization, and we therefore interpret the data as [Ir(**L1**)_2_(en)]Cl cooperatively self‐aggregating in these buffer conditions (25 mM MOPS, pH 7.0, 50 mM NaCl). While it is known that the determination of aggregation numbers from titration calorimetry data is challenging, other thermodynamic parameters are readily available. The CAC of 2.1 µM corresponds to an equilibrium constant for aggregation of 4.8 × 10^5^ M^−1^ and hence a Δ°*G*_aggregation_ of ‐32 kJ mol^−1^. The molar enthalpy for self‐aggregation of −92 kJ mol^−1^ (difference between average pre‐ and post‐CAC heat effects) indicates a highly exothermic self‐aggregation process accompanied by a *T*×Δ°*S*_aggregation_ of + 60 kJ mol^−1^. The aggregation being enthalpy‐driven and entropy‐opposed is in line with “non‐classical” hydrophobic interactions involving extended flat hydrophobic surfaces where solvating water molecules cannot form a fully formed hydrogen‐bond network for geometric reasons, resulting in so‐called dangling hydrogen bonds.^[^ ^44^ ^]^ The release of water molecules with dangling hydrogen bonds from the hydration shells of larger molecules when the larger molecules engage in hydrophobic interactions is therefore enthalpy driven but entropy opposed. We have previously observed similar thermodynamic parameters for the aggregation of flat aromatic H33258 in aqueous solutions.^[^ ^45^ ^]^

Cooperative self‐aggregation of DNA binders in combination with DNA binding makes data analysis complex because of the presence of coupled equilibria.^[^ ^46^ ^]^ In the case of aggregation of charged species, the aggregation process tends to be very sensitive to salt concentration. We therefore studied self‐aggregation in buffer at a lower salt concentration (25 mM MOPS, pH 7.0, 5 mM NaCl) and found no indications of self‐aggregation. We therefore opted to carry out the ITC experiments in a buffer with a reduced salt concentration of 5 mM. Titrations were carried out in two parts, viz. a first titration focussing on the first binding event followed by a second titration focussing on the second binding event (Figures S23–25 with complex and DNA concentrations for each individual titration in Table S5). Figures S23 and S25 clearly show two binding events, as anticipated from UV‐visible titrations.

**Table 5:** DNA‐binding affinities of [Ir(**L**)_2_(en)]Cl in buffer^[^ ^a^ ^]^ at 25 °C according to ITC data.

	[Ir(L1)_2_(en)]Cl	[Ir(L2)_2_(en)]Cl	[Ir(L3)_2_(en)]Cl
*K*_A1_ / 10^6^ M^−1^	239	132	19
*n*_A1_ / bp^−1^	0.06	0.03	0.07
Δ*H*_A1_ / kJ mol^−1^	−3.3	0.6	0.0
*‐T*Δ*S*_A1_ / kJ mol^−1^	−44.5	−46.9	−41.6
*K*_B1_ / 10^6^ M^−1^	0.14	0.41	0.13
*n*_B1_	0.46	0.57	0.31
Δ*H*_B1_ / kJ mol^−1^	−15.6	−12.5	−15.0^[^ ^b^ ^]^
*‐T*Δ*S*_B1_ / kJ mol^−1^	−13.8	−19.5	−14.2

^[a]^ 25 mM MOPS, 5 mM NaCl, pH 7.0.

^[b]^ Restricted during fitting.

**Figure 9:**
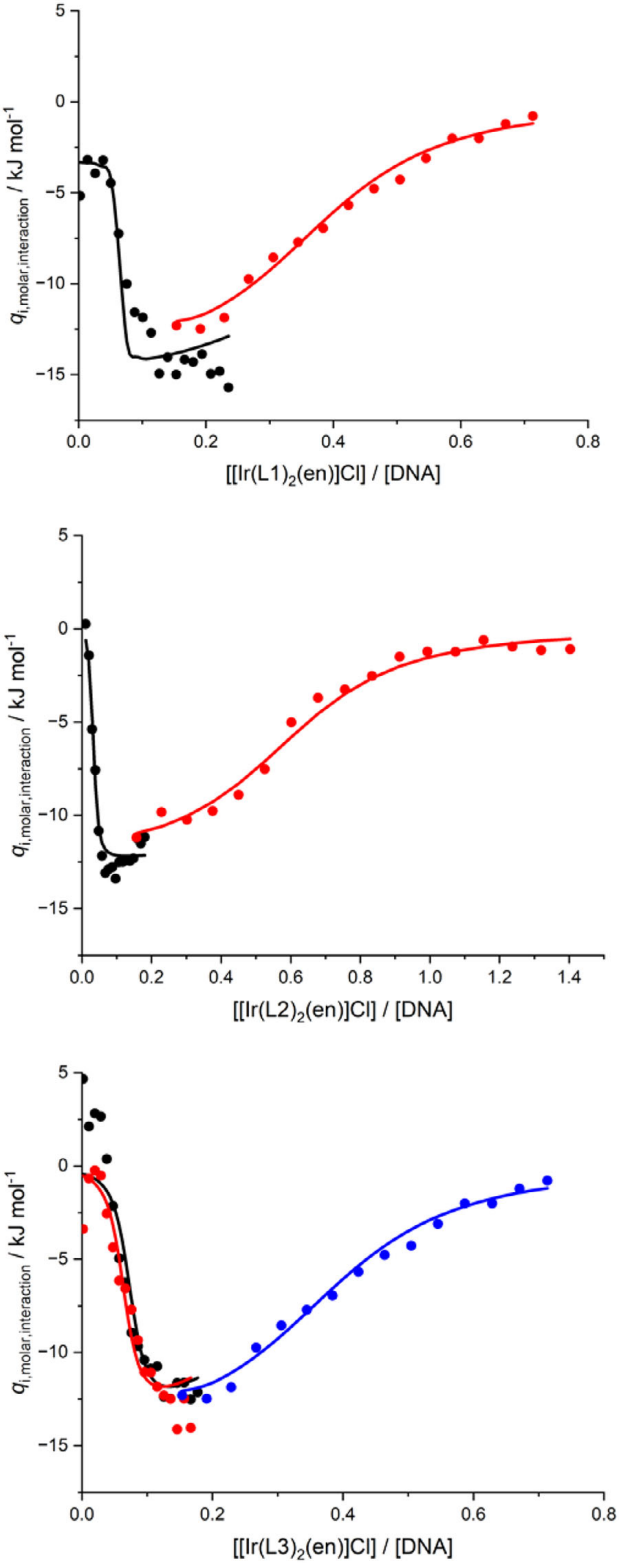
Molar heat effects corrected for heat effects associated with ligand and DNA dilution for the addition of (top‐to‐bottom) [Ir(**L1**)_2_(en)]Cl, [Ir(**L2**)_2_(en)]Cl and [Ir(**L3**)_2_(en)]Cl to DNA.

We analyzed the data using a model involving two types of independent binding sites, correcting for dilution heat effects in our custom data analysis software I2CITC^[^ ^45^, ^47^ ^]^ (Figure 9), and the resulting interaction parameters are summarised in Table 5. The data shows that [Ir(**L1**)_2_(en)]Cl, [Ir(**L2**)_2_(en)]Cl and [Ir(**L3**)_2_(en)]Cl interact with FSDNA in two types of binding sites. The complexes interact with a high‐affinity type binding site with a size of 14–30 basepairs with an affinity *K*_A1_ of the order of 10^7^–10^8^ M^−1^ and negligible enthalpy of interaction. The complexes also bind to a second lower affinity type of binding site with a size of approximately 2–3 basepairs with an affinity *K*_B1_ of the order of 10^5^ M^−1^ in an exothermic process. Based on the combination of binding site size and the exothermic interaction, we attribute the low‐affinity binding event to non‐specific binding of the cationic complexes to negatively charged DNA, supported by hydrophobic interactions between complexes. The binding site size of the higher affinity binding site exceeds the dimensions of the complexes of around five base pairs, as estimated from the docking studies (vide supra). The observed binding site size therefore suggests there may be some selectivity for sequence and/or local DNA structure. Intriguingly, the relatively low Δ*H*_A1_ is in line with groove binding.^[^ ^48^ ^]^

Finally, the influence of DNA binding on the luminescence properties of the complexes was investigated. Steady‐state fluorescence spectra (using *λ*_ex_ = 410 nm) were recorded both in the absence and presence of FSDNA, using a buffer of 25 mM MOPS, pH 7.0, and 50 mM NaCl. First, the appearance and shape of the emission bands (Figure S26) were not influenced by binding to DNA; the position of the emission maxima and the peak shape were generally retained upon the addition of DNA. Overall, the data showed a luminescence intensity enhancement following the addition of DNA, although the relative increases (uncorrected for dilution) varied across the series of complexes; [Ir(**L3**)_2_(en)]Cl and [Ir(**L5**)_2_(en)]Cl showed the largest enhancements of around 40–65%. The observed lifetime values were obtained by fitting the decay curves from pulsed excitation and each showed one emissive species in solution, confirming that all (or at least the vast majority) of the complex is bound to DNA in solution: the lifetime values increase to 0.663, 0.807, 0.415, 1.03, and 0.545 µs for [Ir(**L1‐5**)_2_(en)]Cl, respectively. The time‐resolved luminescence measurements support the inhibition of excited state quenching processes leading to the observed increase in integrated intensity. Interestingly, the most hydrophobic species in the series, [Ir(**L4**)_2_(en)]Cl and [Ir(**L5**)_2_(en)]Cl, showed the largest increases at 75 and 98%, respectively.

## 3 Conclusion

In this study, we were able to develop a series of organometallic cyclometalated Ir(III) species that are compatible with aqueous media. The complexes are phosphorescent in the green region with modest tunability afforded by the use of a single substituent on the phenyl ring of the 2‐phenyl‐benzo[*d*]thiazolyl cyclometalating ligand. The DNA‐binding characteristics of the complexes were studied using combined computational and biophysical experimental approaches. Docking studies predicted that the complexes were unlikely to be intercalators and a groove binding mode is most likely. Experimental studies encompassed UV‐visible titrations and ITC studies. Critically our investigation shows that each of the complexes binds to DNA with apparent affinities in the range 6 × 10^4^ to 4.6 × 10^5^ M^−1^. As part of the ITC investigation, we noted that self‐aggregation is an important consideration and can be alleviated by reducing the salt concentration of the buffer medium. The ITC data showed that for this class of Ir(III) complex there are two types of binding events with FSDNA, viz. a high affinity (10^7^–10^8^ M^−1^) binding site that shows thermodynamics attributed to minor or major groove binding and a low affinity binding site (10^5^ M^−1^) that appears like non‐specific binding of the cationic complexes to negatively charged DNA, supported by hydrophobic interactions between complexes. ITC data and docking studies suggest that binding may take place in the minor or major grooves through H‐bonding interactions. Therefore, the study shows that despite lacking classical features expected for DNA intercalators, these metal complexes, which marry favorable charge and H‐bonding capability, are still effective DNA binders. This may allow the future design of sequence‐selective binders Ir(III) complexes that do not depend upon intercalative modes to drive the DNA interactions.

## 4 Experimental Section

All reactions were performed with the use of a vacuum line and Schlenk techniques. Reagents were commercial grade and were used without further purification. ^1^H, ^19^F, and ^13^C NMR spectra were recorded on a Bruker Avance dpx 500 MHz spectrometer and were recorded on d^6^‐DMSO solutions. ^1^H, ^13^C{^1^H} NMR chemical shifts (δ) were determined relative to internal tetramethylsilane, Si(CH_3_)_4_ and are given in ppm. Mass spectra were obtained by the staff at Cardiff University using a Waters Xevo G2‐XS QTof spectrometer. All photophysical data was obtained on a JobinYvon‐Horiba Fluorolog‐3 spectrometer fitted with a JY TBX picosecond photodetection module. The pulsed source was a Nano‐LED configured for 295 nm output operating at 1 MHz. Luminescence lifetime profiles were obtained using the JobinYvon–Horiba FluoroHub single photon counting module and the data fits yielded the lifetime values using DAS6 deconvolution software. IR spectra were recorded on an ATR‐equipped Shimadzu IRAffinity‐1 spectrophotometer. UV‐vis data were recorded as solutions on a Perkin Elmer Lamda20 spectrophotometer. The pH values of aqueous solutions were determined using a Hanna Instruments pH211 microprocessor pH meter with a Bioblock Scientific pH electrode.

### Method for Ion Exchange

This experiment utilized 30 g of amberlite IRA‐402 ion exchange resin, which underwent a swelling process by immersion in approximately 200 mL of 1 M HCl for three days at around 50 °C. After this swelling procedure, both the resin and the acid were transferred into a column. The column was then purged of the acid by rinsing it with methanol (MeOH) at least five times to ensure the complete removal of any residual acid from the resin. No pressure was applied to the column during packaging or elution. The ion exchange processes were confirmed using ^19^F NMR spectroscopy and elemental analysis.

### Apparent Molar Absorption Coefficients

Initially, 5 mg of each complex was dissolved in 25 mL of dimethyl sulfoxide (DMSO). Subsequently, portions of 2, 4, 6, 8, and 10 mL were withdrawn from the initial solution and transferred into 10 mL volumetric flasks. These flasks were then filled to a total volume of 10 mL with a buffer solution containing 25 mM 3‐(N‐morpholino) propane sulfonic acid (MOPS) and 50 mM sodium chloride (NaCl) at pH 7. The full dissolution of solid materials was confirmed visually by the absence of precipitation. The data for these samples were recorded using UV‐visible spectroscopy, all at a consistent temperature of 298 K.

### UV‐Visible Titrations

All solutions were prepared by dissolving solids in a buffer with stirring, then filtration through a 0.22 µm PES filter to remove solid particles and ensure uniform, saturated solutions before titration. Concentrations were determined using extinction coefficients. MOPS buffer was prepared by dissolving MOPS (3‐(N‐morpholino)propanesulfonic acid, NaCl (all obtained from Fisher and used as supplied) in deionized water (Elga Purelab Flex), adjusting the pH to 7.0 using a NaOH solution (pH of the buffer was determined using a Hanna Instruments pH211 microprocessor pH meter with a Bioblock Scientific pH probe) and making up the solution to 0.5 liter. A stock solution of fish sperm DNA was prepared by dissolving approximately 0.1 g of fish sperm DNA in 10 mL of the buffer. The resulting solution was dialyzed (3.5 kDa MWCO, Visking, Medicell International Ltd) against 0.5 liters of buffer. Following dialysis, the DNA concentration was determined using UV‐visible spectroscopy using a molar absorption coefficient ε_260_ _nm_ = 12 800 M^−1^ cm^−1^.^[^ ^37^ ^]^ Spectra and titrations were plotted in OriginLab Origin 2019b. The titration data were analyzed globally for each complex using an in‐house written version of the multiple independent binding sites model which also explicitly takes changing ligand concentrations into account.^[^ ^49^ ^]^

### Isothermal Calorimetry

Buffers employed in the experiments were 50 mM NaCl and 25 mM MOPS (adjusted to pH 7 with NaOH) and 5 mM NaCl and 25 mM MOPS (adjusted to pH 7 with NaOH). Solutions of DNA were prepared by diluting the DNA stock solutions (see UV‐visible titrations) as required. Complex solutions were prepared by dissolving 5 mg of the complex in 5 mL of buffer, then filtered through a 0.22 µm PES filter to ensure uniform solutions for ITC titration. Concentrations of solutions of complexes and of DNA were determined using UV‐visible spectroscopy. Calorimetric titrations were carried out at 25 °C on a MicroCal PEAQ‐ITC microcalorimeter (Malvern Instruments Ltd., Worcestershire, UK). The instrument was operated by applying a reference power of 10 μcal/s in high‐feedback mode, stirring the sample cell contents at 750 rpm, with a pre‐injection initial delay of 60 s. Solutions of complexes were loaded into the calorimeter injection syringe and DNA solutions were loaded into the sample cell. All experiments involved an initial injection of 0.4 µL in 0.8 s followed by 18 further injections of 2.0 µL in 4.0 s per injection into the calorimeter sample cell. Injections were spaced by at least 90 s to allow full recovery of the baseline. Raw data were treated using MicroCal PEAQ‐ITC Analysis Software (1.41) to generate both integrated heat effects per injection (ΔQ) and molar heat effects per injection (ΔH). Further data analysis was carried out using I2CITC.^[^ ^45^, ^47^ ^]^

### Docking

Docking studies were carried out by using the Autodock Vina 1.1.2 modeling tool.^[^ ^50^ ^]^ The required PDBQT files for the complex structures were generated from the crystal structures for the iridium complexes in which the Ir ion was replaced with a Co ion for compatibility with the docking software. The PDBQT was then generated using AutoDockTools 1.5.6 Sep 17 14.^[^ ^51^ ^]^ The construction of the PDBQT file and the grid box dimensions for the target duplex DNA structure displaying a pre‐formed intercalation gap were described previously.^[^ ^37^ ^]^ The nucleic acid structure was kept rigid in the docking studies and polar hydrogen atoms were added. The top 10 binding modes were generated and ranked by binding affinity. For further parameters, see Table S3. Docked poses were visualized using UCSF Chimera^[^ ^52^ ^]^ after replacing cobalt (as used for compatibility reasons during the docking process) with iridium.

### X‐ray crystallography–Data collection and processing

Suitable crystals of [Ir(**L1**)_2_(en)]PF_6_, [Ir(**L2**)_2_(en)]PF_6_ and [Ir(**L4**)_2_(en)]PF_6_ were selected and data collected following a standard method.^[^ ^53^ ^]^ For each compound the selected crystal was mounted on a MITIGEN holder in oil on a Rigaku FRE + diffractometer with either HF Varimax confocal mirrors ([Ir(**L1**)_2_(en)]PF_6_ and [Ir(**L2**)_2_(en)]PF_6_) or Arc)Sec VHF Varimax confocal mirrors ([Ir(**L4**)_2_(en)]PF_6_), a UG2 goniometer and HyPix 6000HE detector. Each crystal was kept at a steady *T* = 100(2) K during data collection. The structures were solved with the ShelXT^[^ ^54^ ^]^ structure solution program using the Intrinsic Phasing solution method and by using Olex2^[^ ^55^ ^]^ as the graphical interface, the models were refined with either ShelXL^[^ ^56^ ^]^ ([Ir(**L2**)_2_(en)]PF_6_ and [Ir(**L4**)_2_(en)]PF_6_) or using Olex2.refine 1.5^[^ ^57^ ^]^ ([Ir(**L1**)_2_(en)]PF_6_).

CCDC 2 394 742–2394744 contains supplementary X‐ray crystallographic data for [Ir(**L1**)_2_(en)]PF_6_, [Ir(**L2**)_2_(en)]PF_6_ and [Ir(**L4**)_2_(en)]PF_6_ respectively. This data can be obtained free of charge via http://www.ccdc.cam.ac.uk/conts/retrieving.html, or from the Cambridge Crystallographic Data Centre, Union Road, Cambridge, CB2 1EZ; or email: deposit@ccdc.cam.ac.uk.

### Synthesis–General synthetic procedure for the iridium (III) complexes

IrCl_3_.xH_2_O (assumed trihydrate) (0.70 mmol, 1.0 eq.) and the 2‐phenyl‐benzo[*d*]thiazole ligand derivative (1.40 mmol, 2.0 eq.) were added to a Schlenk flask under nitrogen atmosphere. The solution was sparged with N_2_ for 15 minutes following the addition of 10 mL of 2‐ethoxyethanol/H_2_O (3:1). The reaction was heated to reflux for 74 hours, then cooled, and water was added. After filtering the reaction mixture, the crude solid was washed with water and dried under a vacuum to obtain the crude iridium dimer.

The iridium dimer (0.069 mmol, 1.0 eq) and ethylenediamine (en) (excess) were then added to a Schlenk flask with 5 mL of 2‐ethoxyethanol, and the mixture was sparged with N_2_ for 10 minutes. After 36 hours of heating at reflux, the reaction was cooled and 0.1 M NH_4_PF_6_ was added. The resultant precipitate was filtered, washed with water, and dried in vacuo. The crude product was purified by recrystallization using a DCM/MeOH mixture.

***[Ir(2‐phenylbenzo[d]thiazole)][PF_6_]* (*R = H*)**. Isolated as a light orange solid (yield = 400 mg, 84%). ^1^H NMR (500 MHz, d^6^‐DMSO) δ: 8.32 (dd, *J*_HH_ = 1.8, 8.0 Hz, 2H), 7.95 (d, *J*_HH_ = 8.0 Hz, 2H), 7.80 (d, *J*_HH_ = 7.6 Hz, 2H), 7.63‐ 7.57 (m, 4H), 6.90 (dt, *J*_HH_ = 1.4, 7.3 Hz, 2H), 6.68 (dt, *J*_HH_ = 1.3, 7.5 Hz, 2H), 6.36 (d, *J*_HH_ = 7.6 Hz, 2H), 5.32 (d, *J*_HH_ = 6.0 Hz, 2H, N*H*_2_), 4.39‐ 4.36 (br, 2H, N*H*_2_), 2.86–2.84 (br, 2H, C*H*_2_), 2.49–2.43 (br, 2H, C*H*_2_) ppm; ^13^C{^1^H} NMR (126 MHz, d^6^‐DMSO) δ: 182.0 (C = N), 151.5 (C–N), 149.9, 141.0 (C‐S), 134.1, 131.6, 130.4, 127.7, 125.8, 125.6, 123.9, 121.3, 119.3, 45.2 (CH_2_) ppm. FTIR (solid, ATR) *ν*_max_ /cm^− 1^: 3354, 1602, 1581, 1436, 1263, 1124, 1047, 833, 756, 557, 403. HR MS (ES+): *m*/*z* calc'd 673.1072 for C_28_H_24_IrN_4_S_2_; found 673.1063 [M – PF_6_]^+^. Elemental analysis: found, C 40.89%, H 3.00%, N 6.66%; calculated for C_28_H_24_IrN_4_S_2_PF_6_, C 41.12%, H 2.96%, N 6.85%.

***[Ir(2‐(p‐tolyl)benzo[d]thiazole)2(ethane‐1,2‐diamine)][PF_6_] (R = Me)***. Isolated as an orange‐brown solid (yield = 432 mg, 76%). ^1^H NMR (500 MHz, d^6^‐DMSO) δ: 8.28–8.24 (m, 2H), 7.92–7.88 (m, 2H), 7.68 (d, *J*_HH_ = 7.6 Hz, 2H), 7.60–7.55 (m, 4H), 6.73 (dq, *J*_HH_ = 2.4, 7.7 Hz, 2H), 6.16 (s, 2H), 5.26 (d, *J*_HH_ = 6.9 Hz, 2H, N*H*_2_), 4.29–4.28 (br, 2H, N*H*_2_), 2.83–2.82 (br, 2H, C*H*_2_), 2.42 (br, 2H, C*H*_2_), 1.90 (s, 6H, C*H*_3_) ppm; ^13^C{^1^H} NMR (126 MHz, d^6^‐DMSO) δ: 181.9 (C = N), 151.9 (C–N), 150.0, 140.3 (C–S), 138.8, 134.9, 131.5, 127.8, 125.9, 125.5, 123.9, 122.7, 119.2, 45.3 (CH_2_), 21.7 (CH_3_) ppm. FTIR (solid, ATR) *ν*_max_ /cm^− 1^: 3354, 2976, 1600, 1583, 1452, 1236, 1205, 1136, 829, 763, 557, 418. HR MS (ES+): *m*/*z* calc'd 701.1385 for C_30_H_28_IrN_4_S_2_; found 701.1382 [M – PF_6_]^+^. Elemental analysis: found, C 41.61%, H 3.42%, N 6.91%; calculated for C_30_H_28_IrN_4_S_2_PF_6_.H_2_O C 41.71%, H 3.50%, N 6.49%.

***[Ir(2‐(4‐methoxyphenyl)benzo[d]thiazole)2(ethane‐1,2‐diamine)][PF_6_] (R = OMe)***. Isolated as a yellow beige solid (yield = 476 mg, 83%). ^1^H NMR (500 MHz, d^6^‐DMSO) δ: 8.25–8.22 (m, 2H), 7.87–7.85 (m, 2H), 7.76 (d, *J*_HH_ = 8.4 Hz, 2H), 7.58–7.53 (m, 4H), 6.54 (dd, *J*_HH_ = 2.4, 8.5 Hz, 2H), 5.80 (d, 2H), 5.27 (d, *J*_HH_ = 6.3 Hz, 2H, N*H*_2_), 4.36 (br, 2H, N*H*_2_), 3.34 (s, 6H, O–C*H*_3_), 2.86 (br, 2H, C*H*_2_), 2.47–2.44 (br, 2H, C*H*_2_) ppm; ^13^C{^1^H} NMR (126 MHz, d^6^‐DMSO) δ: 181.3 (C = N), 160.8 (C–O), 153.7 (C–N), 150.0 (C–S), 134.4, 131.3, 127.8, 127.7, 125.2, 123.8, 119.3, 119.0, 107.3, 54.4 (CH_3_), 45.4 (CH_2_) ppm. FTIR (solid, ATR) *ν*_max_ /cm^− 1^: 3342, 3267, 1577, 1550, 1423, 1269, 1217, 1035, 831, 754, 555, 405. HR MS (ES+): *m*/*z* calc'd 733.1283 for C_30_H_28_IrN_4_O_2_S_2_; found 733.1281 [M – PF_6_]^+^. Elemental analysis: found, C 40.30%, H 3.50%, N 6.38%; calculated for C_30_H_28_IrN_4_O_2_S_2_PF_6_.H_2_O C 40.22%, H 3.38%, N 6.25%.

***[Ir(2‐(4‐chlorophenyl)benzo[d]thiazole)2(ethane‐1,2‐diamine)][PF_6_] (R = Cl)***. Isolated as a greenish yellow powder (yield = 563 mg, 76%). ^1^H NMR (500 MHz, d^6^‐DMSO) δ: 8.39‐ 8.35 (m, 2H), 7.97–7.93 (m, 2H), 7.89 (d, *J*_HH_ = 8.1 Hz, 2H), 7.67‐ 7.61 (m, 4H), 7.01 (dd, *J*_HH_ = 2.1, 8.2 Hz, 2H), 6.23 (d, 2H), 5.84 (d, *J*_HH_ = 6.8 Hz, 2H, N*H*_2_), 4.55 (br, 2H, N*H*_2_), 2.83 (br, 2H, C*H*_2_), 2.40 (br, 2H, C*H*_2_) ppm; ^13^C{^1^H} NMR (126 MHz, d^6^‐DMSO) δ: 181.1 (C = N), 153.2 (C‐N), 149.5, 140.1 (C‐S), 135.4, 133.1, 131.8, 128.0, 127.6, 126.0, 124.2, 121.8, 119.2, 45.2 (CH_2_) ppm. FTIR (solid, ATR) *ν*_max_ /cm^− 1^: 3354, 1600, 1568, 1421, 1263, 1085, 1043, 955, 833, 754, 557, 443, 410. HR MS (ES+): *m*/*z* calc'd 741.0292 for C_28_H_22_Cl_2_IrN_4_S_2_; found 741.0311 [M – PF_6_]^+^. Elemental analysis: found, C 37.45%, H 2.54%, N 6.28%; calculated for C_28_H_22_Cl_2_IrN_4_S_2_PF_6_, C 37.93%, H 2.50%, N 6.32%.

***[Ir(2‐(4‐(trifluoromethoxy)phenyl)benzo[d]thiazole)2(ethane‐1,2‐diamine)][PF_6_] (R = OCF_3_)***. Isolated as a brown solid (yield = 513 mg, 77%). ^1^H NMR (500 MHz, d^6^‐DMSO) δ: 8.37 (d, *J*_HH_ = 8.2 Hz, 2H), 7.99 (d, *J*_HH_ = 8.0 Hz, 2H), 7.95 (d, *J*_HH_ = 8.0 Hz, 2H), 7.67–7.60 (m, 4H), 6.89 (d, *J*_HH_ = 7.8 Hz, 2H), 6.10 (s, 2H), 5.50–5.47 (br, 2H, NH_2_), 4.60‐ 4.58 (br, 2H, N*H*_2_), 2.86–2.84 (br, 2H, C*H*_2_), 2.44–2.40 (br, 2H, C*H*_2_) ppm; ^13^C{^1^H} NMR (126 MHz, d^6^‐DMSO) δ: 180.8 (C = N), 153.6 (C–O), 149.3, 140.1, 131.8, 127.8 (q, ^2^ *J*_C‐F _= 32.0 Hz), 126.0, 124.2 (q, ^2^ *J*_C‐F _= 28.4 Hz), 119.2 (q, ^1^ *J*_C‐F _= 260.0 Hz), 119.2, 113.6, 45.2 (CH_2_) ppm; ^19^F{^1^H} NMR (471 MHz, d^6^‐DMSO) δ: ‐56.27 (s, 3F, CF_3_). FTIR (solid, ATR) *ν*_max_ /cm^− 1^: 3352, 1585, 1427, 1261, 1041, 997, 833, 754, 557, 401. HR MS (ES+): *m*/*z* calc'd 841.0718 for C_30_H_22_F_6_IrN_4_O_2_S_2_; found 841.0718 [M – PF_6_]^+^. Elemental analysis: found, C 36.68%, H 2.31%, N 5.87%; calculated for C_30_H_22_F_6_IrN_4_O_2_S_2_PF_6_, C 36.55%, H 2.25%, N 5.68%.

## Conflict of Interests

The authors declare no conflict of interest.

## Supporting information



Supporting information
