# Supplementary material for: DNA‐Binding Properties of Non‐Intercalating Water‐Soluble Organometallic Ir(III) Luminophores
Source: Chemistry. 2025 May 2;31(30):e202500290. doi: 10.1002/chem.202500290 (PMC12117180; doi:10.1002/chem.202500290)
Supplement: Supplementary file 1 — Supporting information [file CHEM-31-e202500290-s001.pdf]

Supplementary Information

DNA-binding properties of non-intercalating organometallic Ir(III) luminophores

Ibrahim S. Alkhaibari,[a,b] Peter N. Horton,[c] Simon J. Coles,[c] Niklaas J. Buurma,[a]* and Simon J. A. Pope[a]*

[a]School of Chemistry, Main Building, Cardiff University, Cardiff CF10 3AT, Cymru/Wales; [b] Department of Chemistry, College of Science, Qassim University, Buraydah 52571, Saudi Arabia; [c] UK National Crystallographic Service, Chemistry, Faculty of Natural and Environmental Sciences, University of Southampton, Highfield, Southampton, SO17 1BJ, England, UK.

Email: buurma@cardiff.ac.uk; popesj@cardiff.ac.uk

Contents

	Experimental procedures for the ligands (**L1-L5**) and associated characterization data	3-4
Figure S1	^{1}H NMR spectrum of [Ir(**L1**)$_2$(en)][PF$_6$]	5
Figure S2	^{13}C{^{1}H} NMR spectrum of [Ir(**L1**)$_2$(en)][PF$_6$]	5
Figure S3	^{1}H NMR spectrum of [Ir(**L2**)$_2$(en)][PF$_6$]	6
Figure S4	^{13}C{^{1}H} NMR spectrum of [Ir(**L2**)$_2$(en)][PF$_6$]	6
Figure S5	^{1}H NMR spectrum of [Ir(**L3**)$_2$(en)][PF$_6$]	7
Figure S6	^{13}C{^{1}H} NMR spectrum of [Ir(**L3**)$_2$(en)][PF$_6$]	7
Figure S7	^{1}H NMR spectrum of [Ir(**L4**)$_2$(en)][PF$_6$]	8
Figure S8	^{13}C{^{1}H} NMR spectrum of [Ir(**L4**)$_2$(en)][PF$_6$]	8
Figure S9	^{1}H NMR spectrum of [Ir(**L5**)$_2$(en)][PF$_6$]	9
Figure S10	^{13}C{^{1}H} NMR spectrum of [Ir(**L5**)$_2$(en)][PF$_6$]	9
Figure S11	HRMS data for the five Ir(III) complexes	10
Figure S12	IR spectra (solid state) for the five Ir(III) complexes	11
Figure S13	Computed docking of **L1** with an open d(ATCGAGACGTCTCGAT)$_2$ structure	14
Figure S14	Graphical plots used (in triplicate) for the molar absorption coefficient calculation of [Ir(**L1**)$_2$(en)]Cl	15
Figure S15	Graphical plots used (in triplicate) for the molar absorption coefficient calculation of [Ir(**L2**)$_2$(en)]Cl	15
Figure S16	Graphical plots used (in triplicate) for the molar absorption coefficient calculation of [Ir(**L3**)$_2$(en)]Cl	15

Figure S17	Graphical plots used (in triplicate) for the molar absorption coefficient calculation of [Ir(**L4**)$_2$(en)]Cl	16
Figure S18	Graphical plots used (in triplicate) for the molar absorption coefficient calculation of [Ir(**L5**)$_2$(en)]Cl	16
Figure S19	Fitted UV-vis titration data for [Ir(**L1**)$_2$(en)]Cl (44.14 μM) with sequential aliquots of FSDNA (25 mM MOPS, 5 mM NaCl, pH 7.00, at 25 °C)	17
Figure S20	Fitted UV-vis titration data for [Ir(**L2**)$_2$(en)]Cl (35.17 μM) with sequential aliquots of FSDNA (25 mM MOPS, 5 mM NaCl, pH 7.00, at 25 °C)	18
Figure S21	Fitted UV-vis titration data for [Ir(**L4**)$_2$(en)]Cl (29.62 μM) with sequential aliquots of FSDNA (25 mM MOPS, 5 mM NaCl, pH 7.00, at 25 °C)	19
Figure S22	Fitted UV-vis titration data for [Ir(**L5**)$_2$(en)]Cl (37.55 μM) with sequential aliquots of FSDNA (25 mM MOPS, 5 mM NaCl, pH 7.00, at 25 °C)	20
Figure S23	ITC data obtained for the addition of [Ir(**L1**)$_2$(en)]Cl to DNA showing two distinct binding events	21
Figure S24	ITC data obtained for the addition of [Ir(**L2**)$_2$(en)]Cl to DNA showing two distinct binding events	22
Figure S25	ITC data obtained for the addition of [Ir(**L3**)$_2$(en)]Cl to DNA showing two distinct binding events	22
Figure S26	Photoluminescence steady state data before and after addition of FS DNA (recorded in MOPS and 5 mM NaCl with a pH of 7.00 at 298 K)	23

Table S1	The data collection parameters from the X-ray crystallography	12
Table S2	Selected bond angles for the X-ray structures	13
Table S3	Calculated molar absorption coefficients for the complexes in MOPS buffer and 50 mM sodium chloride (NaCl) at pH 7	13
Table S4	Parameters used in docking studies	13
Table S5	Sample conditions for the isothermal calorimetric measurements	21

Synthesis of 2-phenylbenzo[d]thiazole (L1). White; (900 mg, 95%) ^{1}H NMR (500 MHz, DMSO) δ: 8.15 (d, J_{HH} = 8.1 Hz, 1H, H_{arom}), 8.11- 8.09 (m, 2H, H_{arom}), 8.07 (d, J_{HH} = 8.0 Hz, 1H, H_{arom}), 7.60- 7.57 (m, 3H, H_{arom}), 7.55 (d, J_{HH} = 7.0 Hz, 1H, H_{arom}), 7.47 (td, J_{HH} = 1.2, 6.0 Hz, 1H, H_{arom}) ppm; ^{13}C NMR (126 MHz, DMSO) δ: 167.3 (C=N), 153.5 (C-N), 134.4 (C-S), 132.8, 131.4, 129.4, 127.2, 126.6, 125.5, 122.9, 122.3 ppm. FTIR (solid, ATR) v_{max} /cm^{-1}: 3064, 1510, 1477, 1433, 1313, 1253, 1224, 1159, 1070, 1028, 962, 758, 729, 686, 623, 549, 466, 418. LR MS (ES^+): *m*/*z* calcd 211.2820 for $C_{13}H_9NS$; found 212.05 $[M + H]^+$.[1]

Synthesis of 2-(p-tolyl)benzo[d]thiazole (L2). Beige; (833 mg, 82%) ^{1}H NMR (500 MHz, DMSO) δ: 8.13 (ddd, J_{HH} = 1.9, 8.0 Hz, 1H, H_{arom}), 8.04 (ddd, J_{HH} = 1.8, 8.1 Hz, 1H, H_{arom}), 7.98 (t, J_{HH} = 2.1, 8.1 Hz, 2H, H_{arom}), 7.53 (td, J_{HH} = 1.2, 7.1 Hz, 1H, H_{arom}), 7.45 (td, J_{HH} = 1.2, 7.1 Hz, 1H, H_{arom}), 7.38 (dt, J_{HH} = 1.4, 7.8 Hz, 2H, H_{arom}), 2.39 (s, 3H, CH_3) ppm; ^{13}C NMR (126 MHz, DMSO) δ: 167.3 (C=N), 153.5 (C-N), 141.5 (C-CH_3), 134.3 (C-S), 130.2, 129.9, 127.1, 126.6, 125.3, 122.7, 122.3, 21.0 (CH_3) ppm. FTIR (solid, ATR) v_{max} /cm^{-1}: 3057, 2837, 1606, 1481, 1433, 1311, 1251, 1226, 1182, 1111, 1010, 962, 817, 766, 729, 692, 623, 551, 484. LR MS (ES^+): *m*/*z* calcd 225.3090 for $C_{14}H_{11}NS$; found 226.07 $[M + H]^+$.[1]

Synthesis of 2-(4-methoxyphenyl)benzo[d]thiazole (L3). Beige; (950 mg, 87%) ^{1}H NMR (500 MHz, DMSO) δ: 8.11 (ddd, J_{HH} = 1.9, 7.9 Hz, 1H, H_{arom}), 8.03 (t, J_{HH} = 3.0, 8.9 Hz, 2H, H_{arom}), 8.01 (ddd, J_{HH} = 1.9, 8.1 Hz, 1H, H_{arom}), 7.52 (td, J_{HH} = 1.3, 7.2 Hz, 1H, H_{arom}), 7.42 (td, J_{HH} = 1.3, 7.2 Hz, 1H, H_{arom}), 7.12 (t, J_{HH} = 2.7, 8.3 Hz, 2H, H_{arom}), 3.86 (s, 3H, CH_3) ppm; ^{13}C NMR (126 MHz, DMSO) δ: 167.0 (C=N), 161.8 (C-O), 153.6 (C-N), 134.2 (C-S), 128.8, 126.5, 125.1, 122.4, 122.2, 114.7, 55.5 (CH_3) ppm. FTIR (solid, ATR) v_{max} /cm^{-1}: 2995, 2835, 1604, 1483, 1433, 1309, 1255, 1224, 1170, 1114, 1026, 968, 831, 758, 731, 692, 634, 551, 511, 427. LR MS (EI): *m*/*z* calcd 241.0561 for $C_{14}H_{11}NOS$; found 241.05.[1]

Synthesis of 2-(4-Chlorophenyl)benzothiazole (L4). Beige; (1062 mg, 96%) ^{1}H NMR (500 MHz, DMSO) δ: 8.15 (d, J_{HH} = 7.9 Hz, 1H, H_{arom}), 8.10 (dt, J_{HH} = 2.7, 8.6

Hz, 2H, H_{arom}), 8.10 (d, J_{HH} = 8.1 Hz, 1H, H_{arom}), 7.63 (dt, J_{HH} = 2.3, 8.8 Hz, 2H, H_{arom}), 7.56 (td, J_{HH} = 1.2, 7.2 Hz, 1H, H_{arom}), 7.48 (td, J_{HH} = 1.2, 7.1 Hz, 1H, H_{arom}) ppm; ^{13}C NMR (126 MHz, DMSO) δ: 165.9 (C=N), 153.4 (C-N), 136.0 (C-S), 134.5 (C-Cl), 131.6, 129.4, 128.8, 126.7, 125.7, 122.9, 122.4 ppm. FTIR (solid, ATR) v_{max} /cm^{-1}: 3055, 1589, 1473, 1435, 1398, 1315, 1251, 1089, 1012, 964, 827, 756, 692, 549, 482, 418. LR MS (ES$^+$): *m*/*z* calcd 245.01 for $C_{13}H_8NClS$; found 246.02 [M + H]$^+$.[1]

Synthesis of 2-(4-(trifluoromethoxy)phenyl)benzo[d]thiazole (L5). Beige; (1203 mg, 90%) ^{1}H NMR (500 MHz, DMSO) δ: 8.23 (dt, J_{HH} = 2.7, 8.9 Hz, 2H, H_{arom}), 8.18 (ddd, J_{HH} = 2.1, 8.0 Hz, 1H, H_{arom}), 8.09 (ddd, J_{HH} = 1.9, 8.0 Hz, 1H, H_{arom}), 7.59- 7.56 (m, 3H, H_{arom}), 7.49 (td, J_{HH} = 1.3, 7.1 Hz, 1H, H_{arom}) ppm; ^{13}C NMR (126 MHz, DMSO) δ: 165.6 (C=N), 153.4 (C-N), 150.2 (C-S), 134.7, 131.9, 129.3, 126.8, 125.8, 123.0, 122.5, 121.7, 120.0 (q, $^1J_{C\text{-}F}$ = 255.4 Hz) ppm; ^{19}F NMR (471 MHz, DMSO) δ: -56.67 (s, 3F, CF_3) ppm. FTIR (solid, ATR) v_{max} /cm^{-1}: 3055, 1606, 1517, 1481, 1435, 1309, 1253, 1153, 970, 756, 731, 657, 617, 551, 489. LR MS (ES$^+$): *m*/*z* calcd 295.2792 for $C_{14}H_8F_3NOS$; found 296.06 [M + H]$^+$.[2]

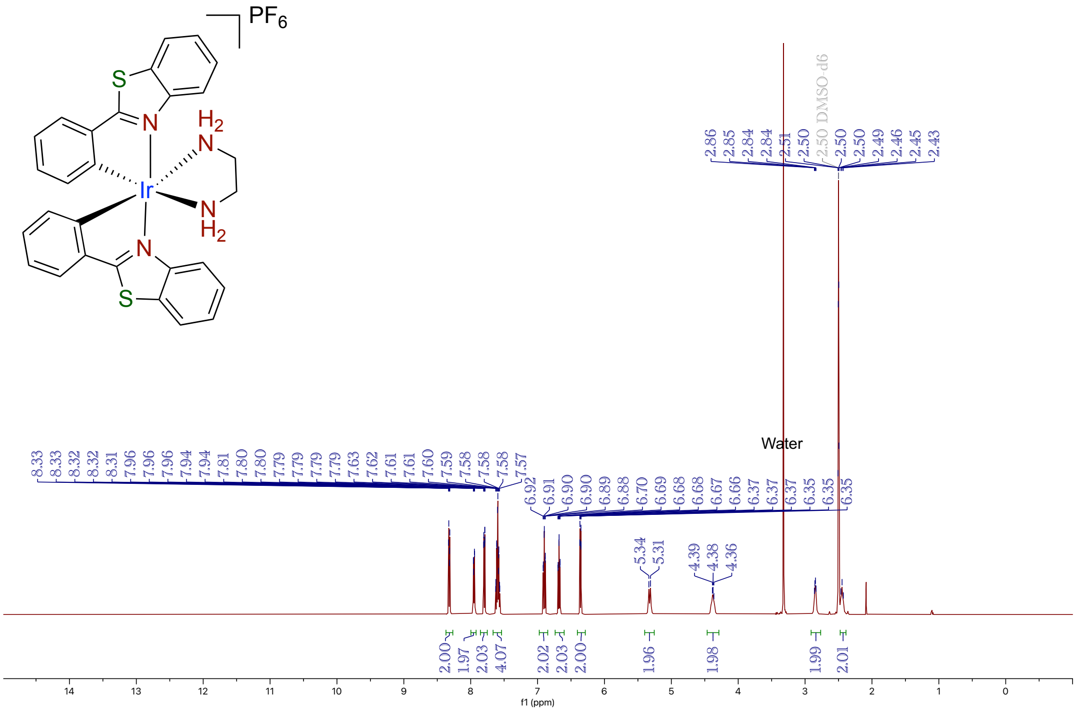

Figure S1. ^{1}H NMR spectrum of $[Ir(L1)_2(en)][PF_6]$

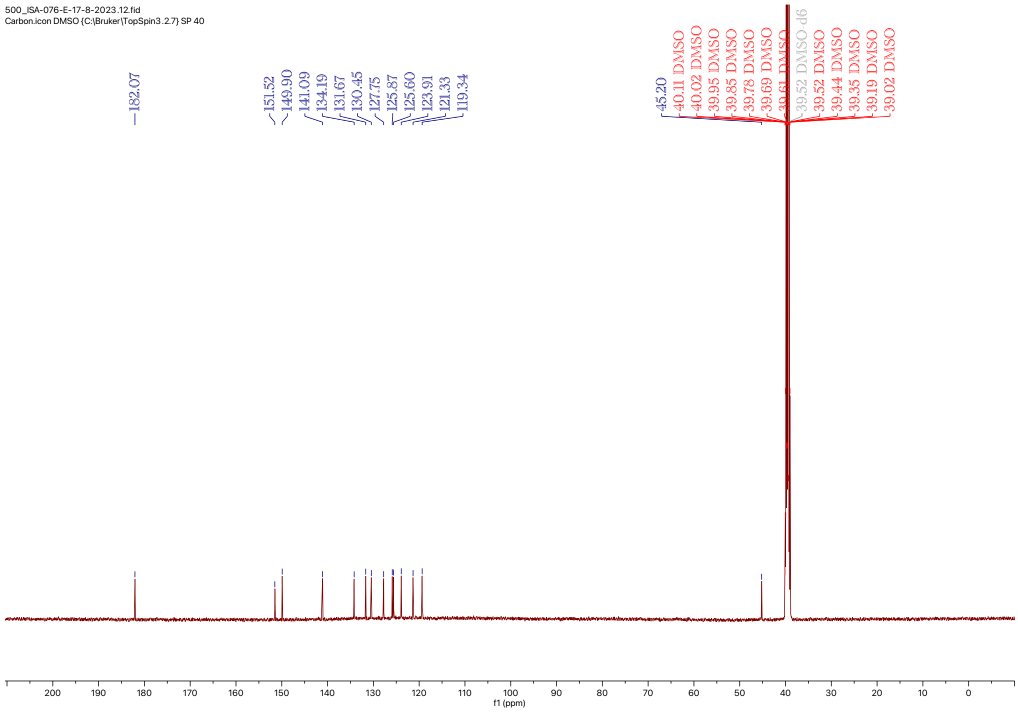

Figure S2. $^{13}C\{^{1}H\}$ NMR spectrum of $[Ir(L1)_2(en)][PF_6]$

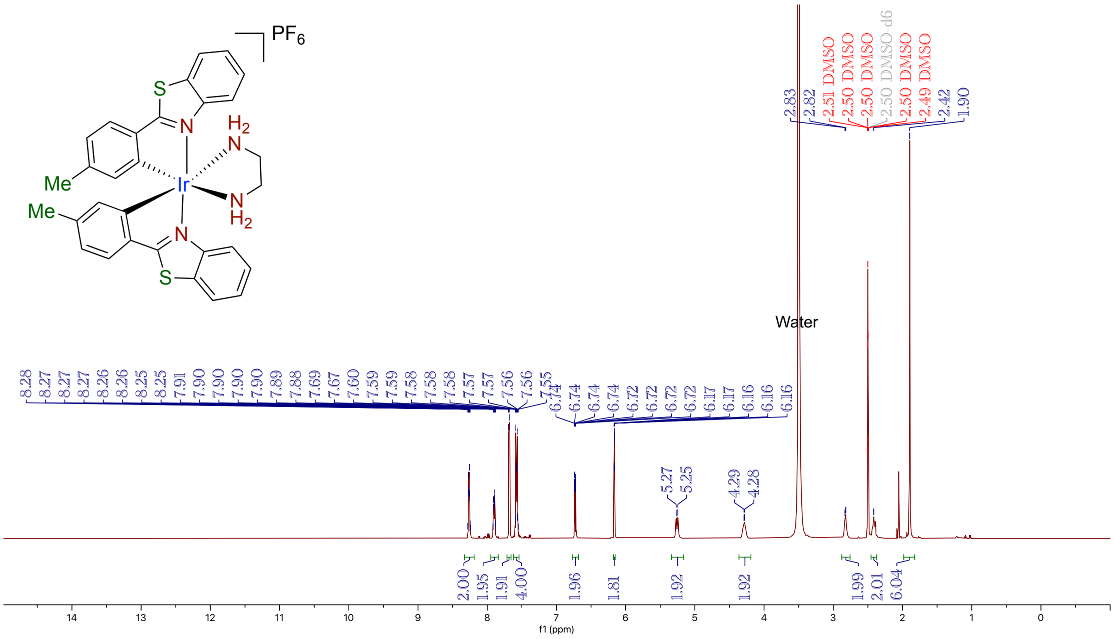

Figure S3. ^{1}H NMR spectrum of $[Ir(L2)_2(en)][PF_6]$

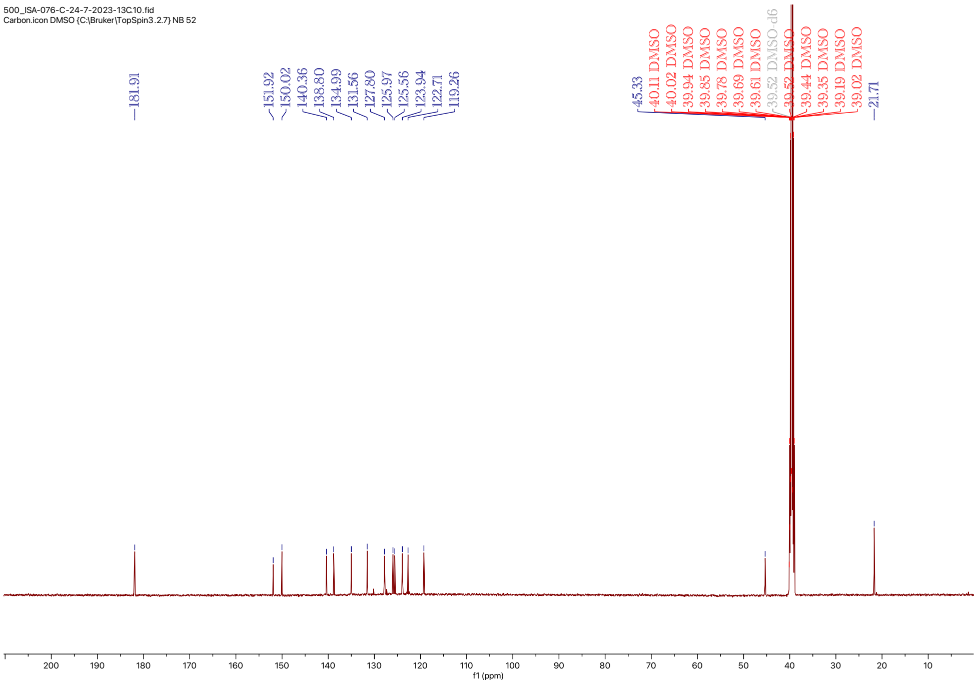

Figure S4. $^{13}C\{^{1}H\}$ NMR spectrum of $[Ir(L2)_2(en)][PF_6]$

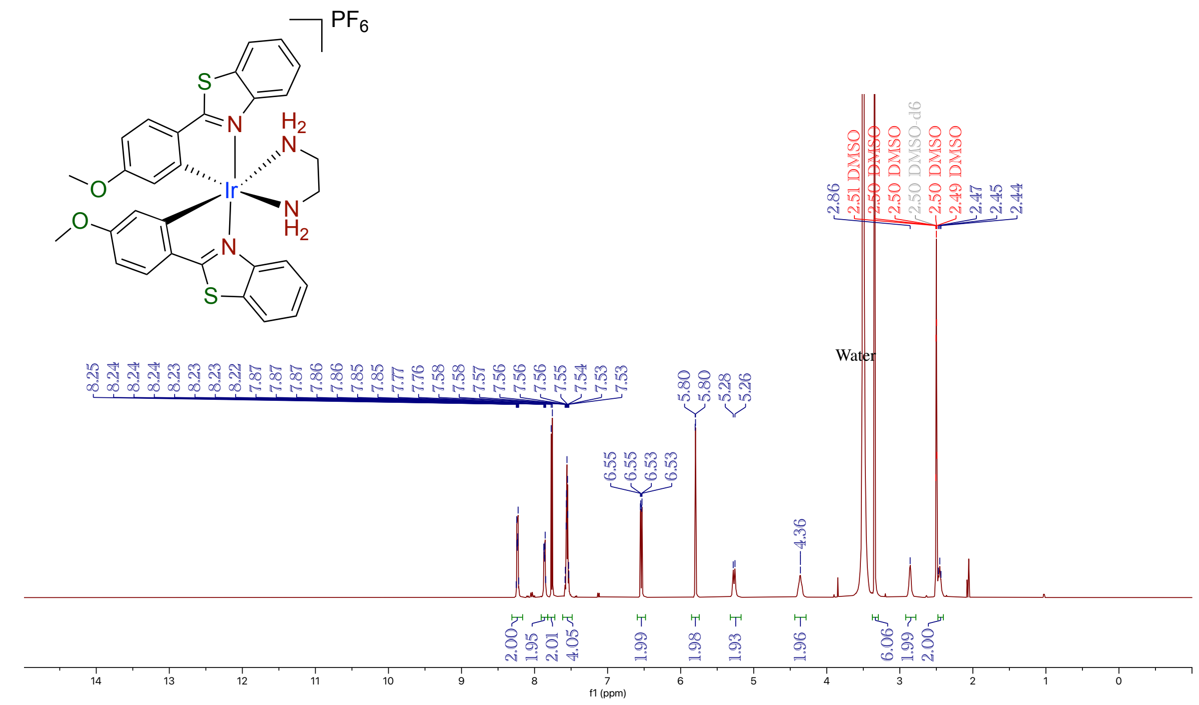

Figure S5. ^{1}H NMR spectrum of $[Ir(L3)_2(en)][PF_6]$

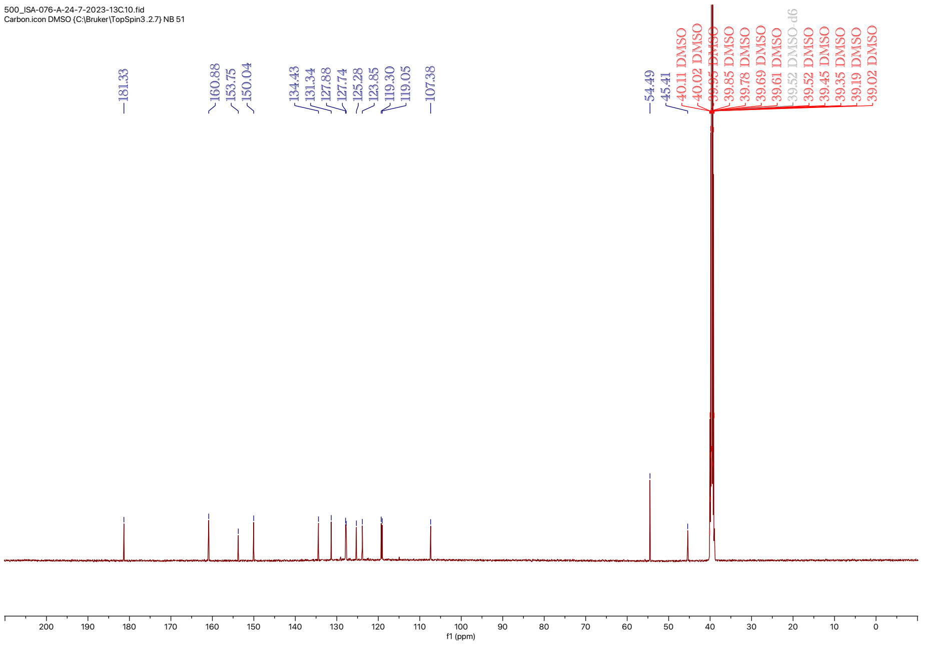

Figure S6. $^{13}C\{^{1}H\}$ NMR spectrum of $[Ir(L3)_2(en)][PF_6]$

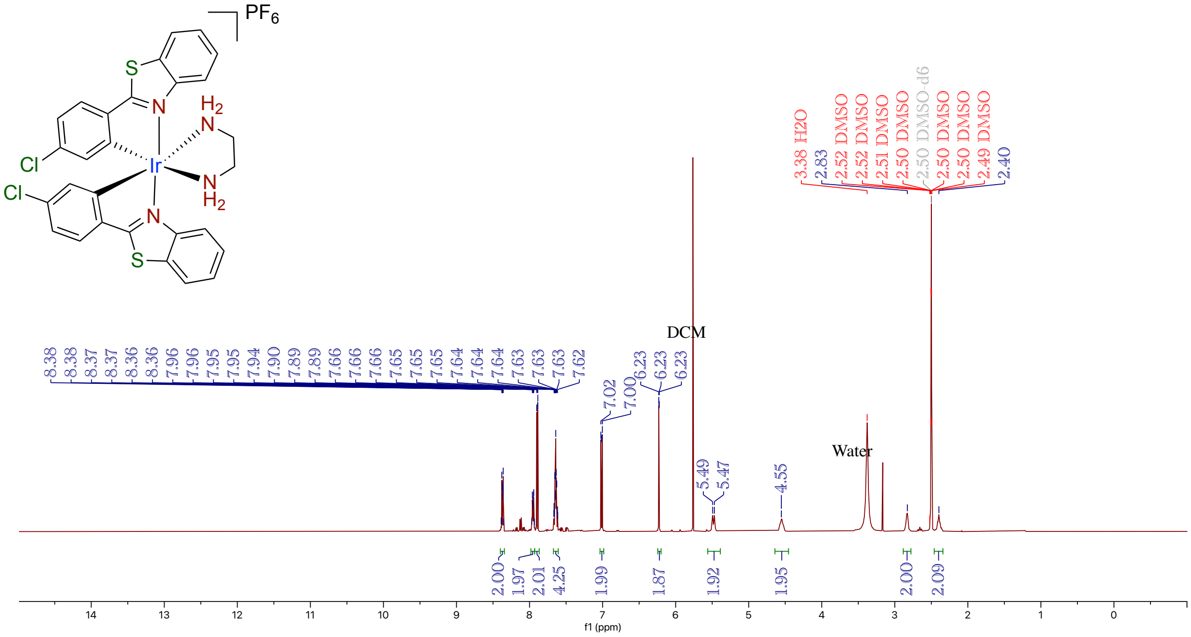

Figure S7. ^{1}H NMR spectrum of [Ir(L4)$_2$(en)][PF$_6$]

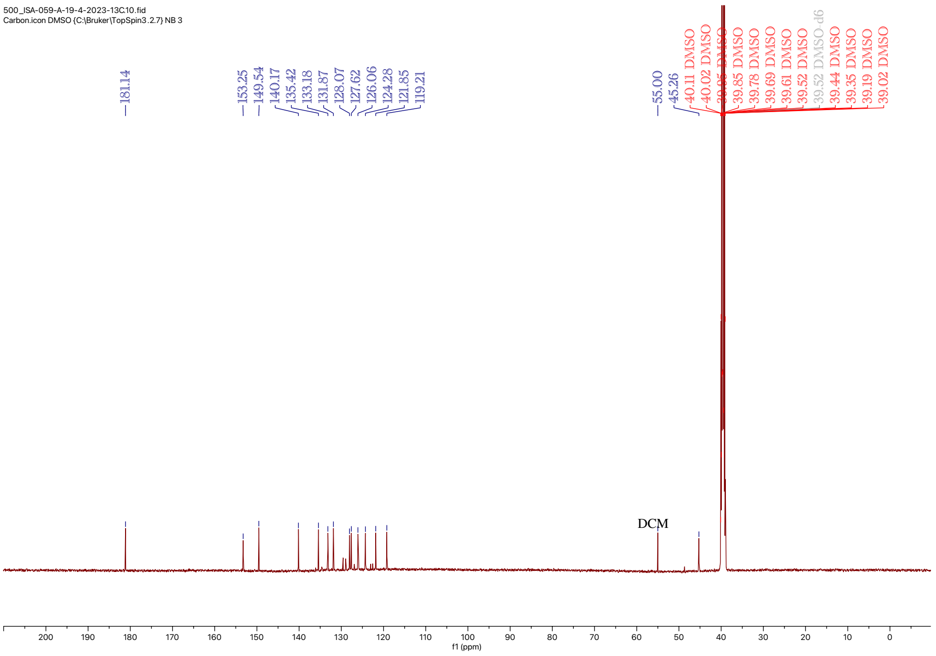

Figure S8. $^{13}C\{^{1}H\}$ NMR spectrum of [Ir(L4)$_2$(en)][PF$_6$]

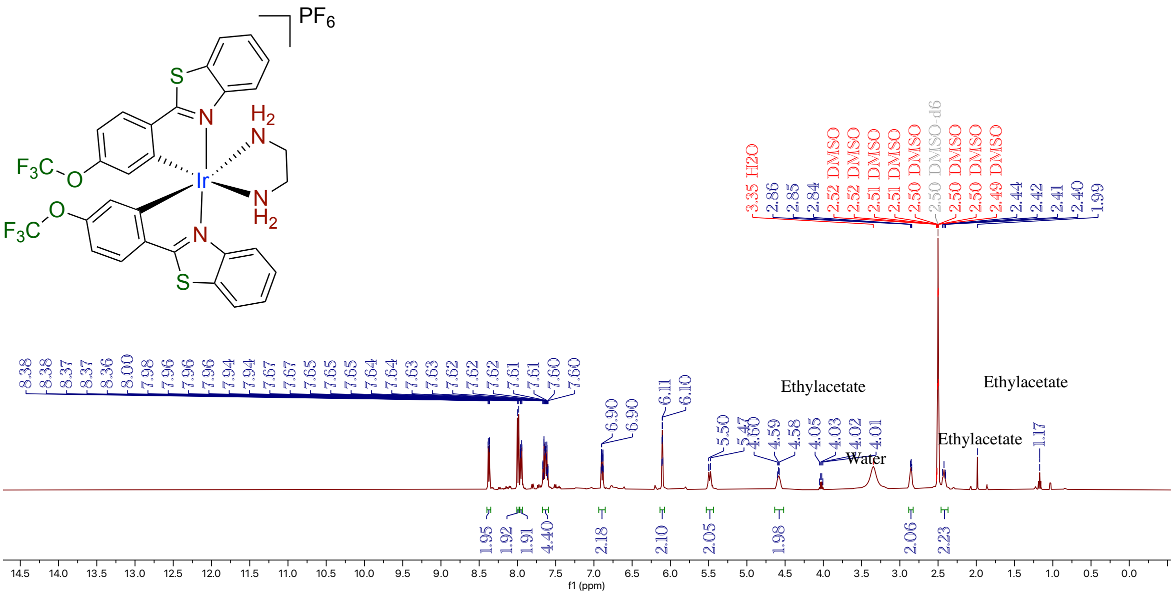

Figure S9. ^{1}H NMR spectrum of $[Ir(L5)_2(en)][PF_6]$

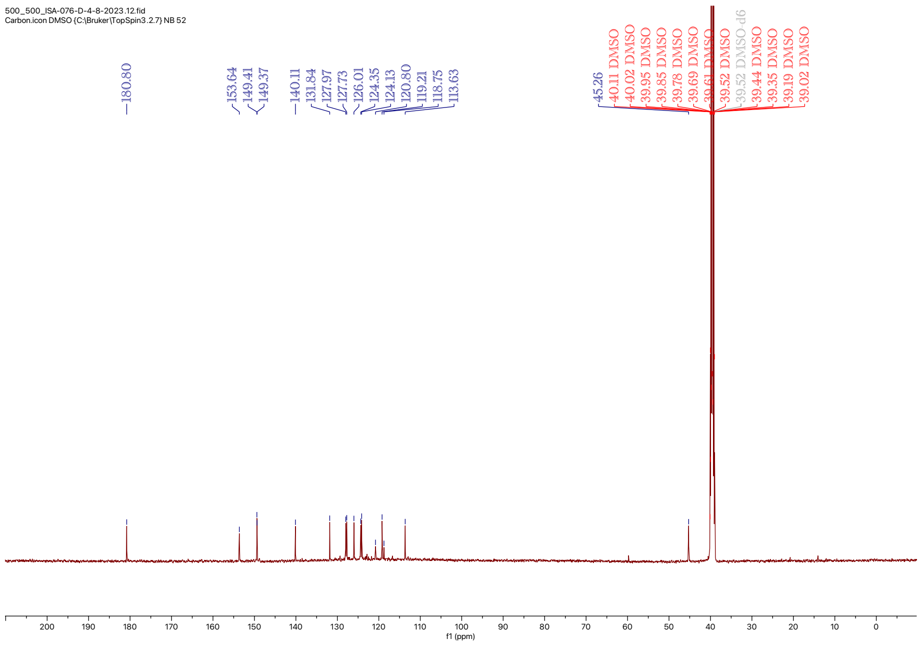

Figure S10. $^{13}C\{^{1}H\}$ NMR spectrum of $[Ir(L5)_2(en)][PF_6]$

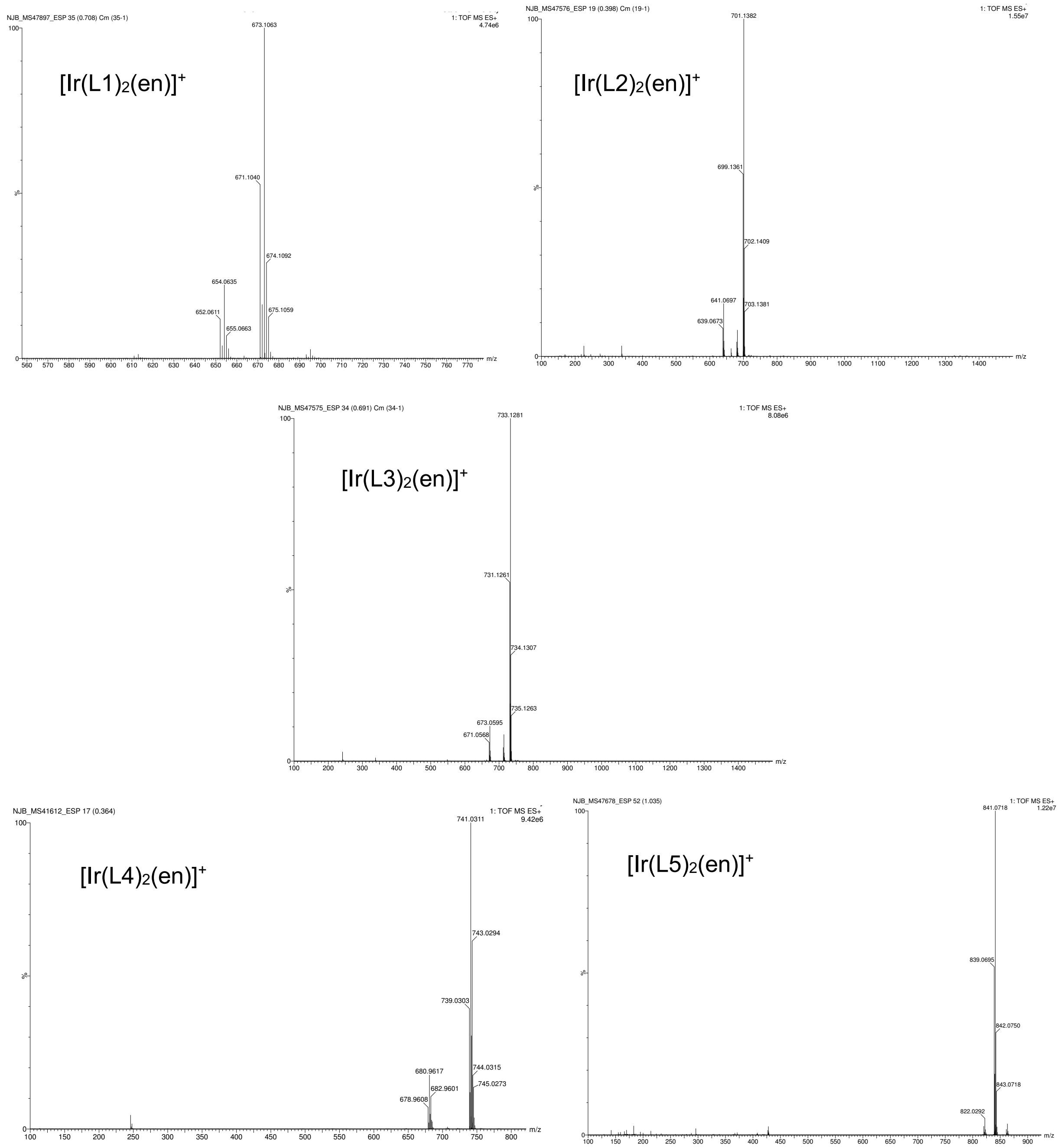

Figure S11. HRMS data for the five Ir(III) complexes.

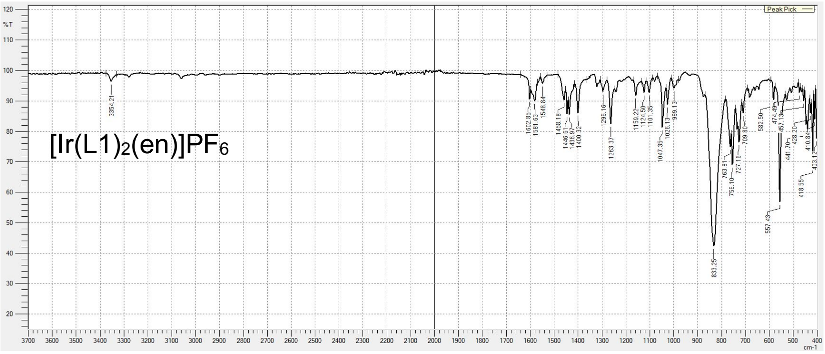

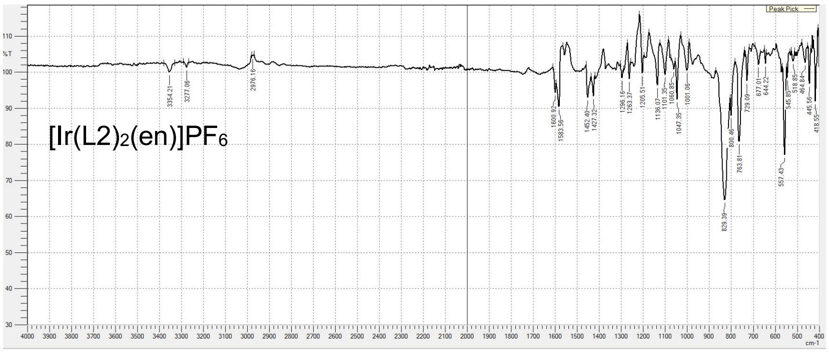

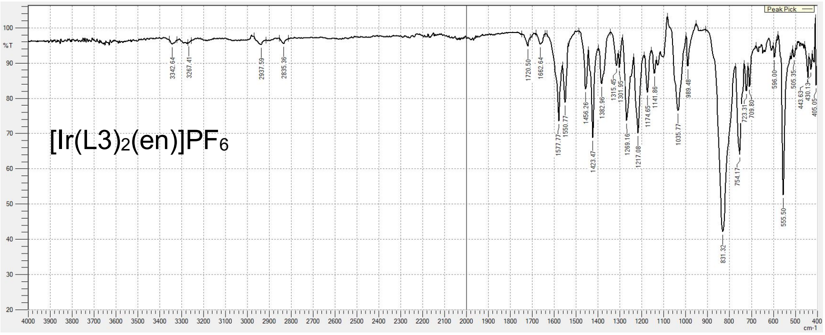

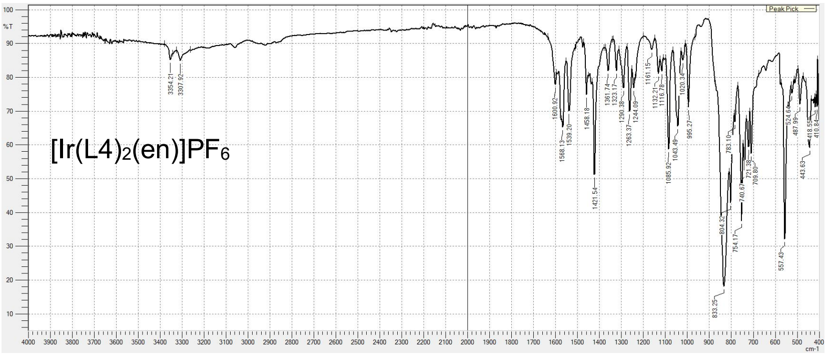

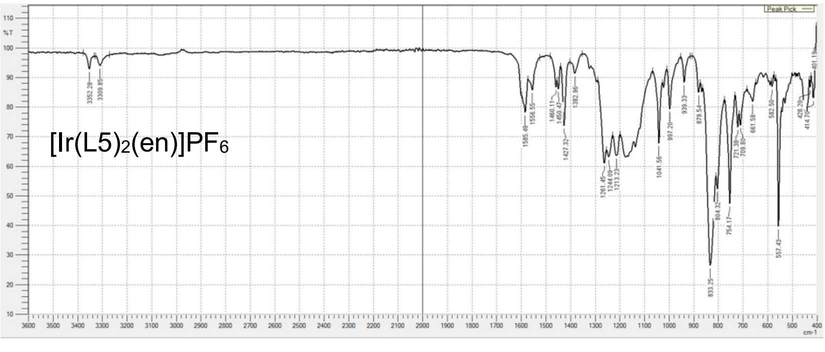

Figure S12. IR spectra (solid state) for the five Ir(III) complexes.

Table S1. The data collection parameters from the X-ray crystallography.

**Compound**	**[Ir(L1)$_2$(en)]PF$_6$**	**[Ir(L2)$_2$(en)]PF$_6$**	**[Ir(L4)$_2$(en)]PF$_6$**
Formula	$C_{32}H_{30}F_6IrN_6PS_2$	$C_{32}H_{31}F_6IrN_5PS_2$	$C_{34}H_{31}Cl_2F_6IrN_7PS_2$
$D_{calc.}$/ g cm^{-3}	1.751	1.706	1.785
μ/mm^{-1}	4.146	4.096	3.916
Formula Weight	899.948	886.91	1009.85
Colour	orange	orange	orange
Shape	plate-shaped	block-shaped	rod-shaped
Size/mm^3	0.252×0.169×0.028	0.220×0.130×0.060	0.220×0.050×0.030
T/K	100(2)	100(2)	100(2)
Crystal System	monoclinic	trigonal	triclinic
Space Group	*Cc*	*R*-3*c*	*P*-1
a/Å	18.7945(1)	37.2230(2)	8.44700(10)
b/Å	11.3260(1)	37.2230(2)	13.89540(10)
c/Å	16.5267(1)	12.95280(10)	16.54480(10)
α/°	90	90	82.7890(10)
β/°	103.941(1)	90	84.3650(10)
γ/°	90	120	77.8930(10)
V/Å^3	3414.36(4)	15542.4(2)	1878.55(3)
Z	4	18	2
Z'	1	0.5	1
Wavelength/Å	0.71075	0.71075	0.71075
Radiation type	Mo K_α	Mo K_α	Mo K_α
Θ_{min}/°	2.12	2.295	2.056
Θ_{max}/°	68.16	43.111	38.088
Measured Refl's.	212703	265519	179655
Indep't Refl's	57696	12876	19797
Refl's I≥2 σ(I)	48096	11249	18626
R_{int}	0.0250	0.0463	0.0536
Parameters	524	238	536
Restraints	393	0	246
Largest Peak	2.6909	1.414	1.725
Deepest Hole	-0.7445	-1.281	-2.604
GooF	1.0027	1.020	1.037
wR_2 (all data)	0.0600	0.0537	0.0477
wR_2	0.0554	0.0521	0.0471
R_1 (all data)	0.0380	0.0311	0.0222
R_1	0.0269	0.0245	0.0197

Table S2. Selected bond angles (°) for the X-ray crystal structures.

**[Ir(L2)$_2$(en)]PF$_6$**				**[Ir(L4)$_2$(en)]PF$_6$**				**[Ir(L1)$_2$(en)]PF$_6$**			
N1	Ir1	N1^1	171.30(6)	N1	Ir1	N41	101.31(4)	N21	Ir1	N1	170.10(4)
N1^1	Ir1	N21^1	85.16(4)	N1	Ir1	N42	85.64(4)	N41	Ir1	N1	101.27(3)
N1	Ir1	N21	85.16(4)	N21	Ir1	N1	170.83(4)	N41	Ir1	N21	86.00(3)
N1^1	Ir1	N21	101.63(4)	N21	Ir1	N41	86.55(4)	N42	Ir1	N1	85.81(3)
N1	Ir1	N21^1	101.63(4)	N21	Ir1	N42	100.59(4)	N42	Ir1	N21	102.28(4)
N21^1	Ir1	N21	78.85(5)	N42	Ir1	N41	79.31(4)	N42	Ir1	N41	79.09(4)
C1	Ir1	N1	79.83(5)	C1	Ir1	N1	79.81(4)	C1	Ir1	N1	79.93(4)
C1^1	Ir1	N1^1	79.83(5)	C1	Ir1	N21	93.13(4)	C1	Ir1	N21	93.86(4)
C1^1	Ir1	N1	94.45(4)	C1	Ir1	N41	171.43(4)	C1	Ir1	N41	171.58(4)
C1	Ir1	N1^1	94.45(4)	C1	Ir1	N42	92.36(4)	C1	Ir1	N42	92.73(4)
C1	Ir1	N21^1	169.88(4)	C1	Ir1	C21	97.94(5)	C21	Ir1	N1	92.88(4)
C1	Ir1	N21	91.35(4)	C21	Ir1	N1	95.16(4)	C21	Ir1	N21	79.99(4)
C1^1	Ir1	N21^1	91.35(4)	C21	Ir1	N21	79.92(4)	C21	Ir1	N41	92.59(4)
C1^1	Ir1	N21	169.88(4)	C21	Ir1	N41	90.43(4)	C21	Ir1	N42	171.13(4)
C1	Ir1	C1^1	98.55(7)	C21	Ir1	N42	169.65(4)	C21	Ir1	C1	95.68(4)

**Table S3**. Parameters used in Autodock Vina docking studies	
DNA structure	open d(ATCGAGACGTCTCGAT)$_2$ predictor_gap_relaxed_ATCGAGACGTCTCGAT.pdbqt (See DOI: 10.1039/c1cc00111f)
centre_x	-2.152
centre_y	2.953
centre_y	24.92
size_x	40
size_y	40
size_z	60
exhaustiveness	200
num_modes	10

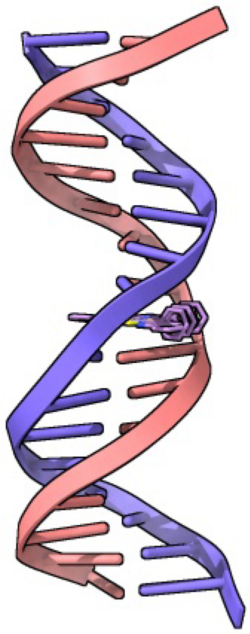

Figure S13. Docking of **L1** with the open d(ATCGAGACGTCTCGAT)$_2$ structure.

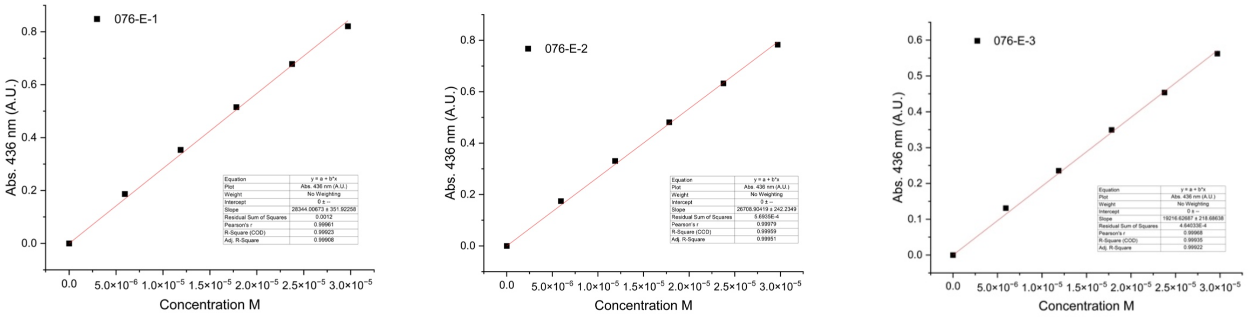

Figure S14. Graphical plots used (in triplicate) for the molar absorption coefficient calculation of [Ir(**L1**)$_2$(en)]Cl in mixtures of buffer (25 mM MOPS and 50 mM sodium chloride at pH 7) and DMSO.

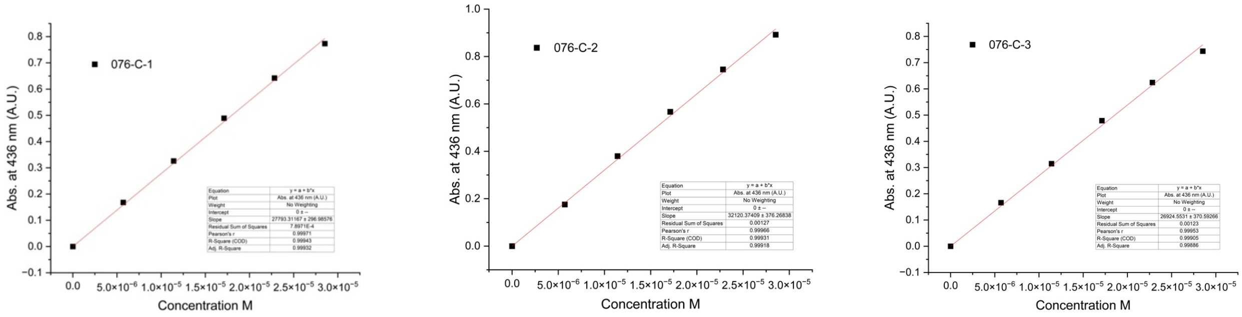

Figure S15. Graphical plots used (in triplicate) for the molar absorption coefficient calculation of [Ir(**L2**)$_2$(en)]Cl in mixtures of buffer (25 mM MOPS and 50 mM sodium chloride at pH 7) and DMSO.

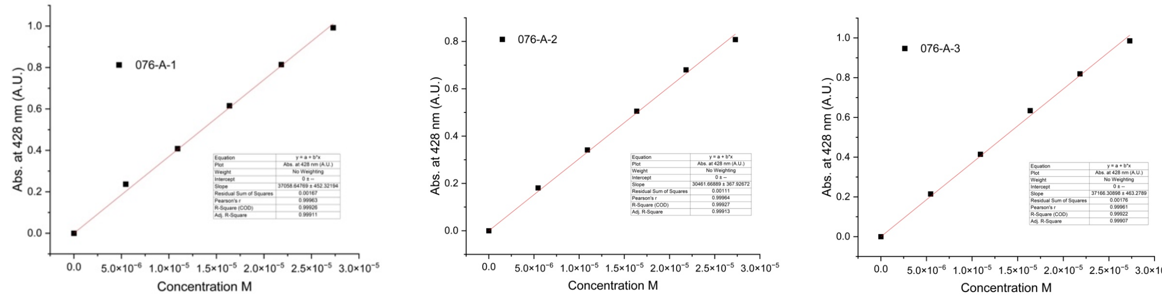

Figure S16. Graphical plots used (in triplicate) for the molar absorption coefficient calculation of [Ir(**L3**)$_2$(en)]Cl in mixtures of buffer (25 mM MOPS and 50 mM sodium chloride at pH 7) and DMSO.

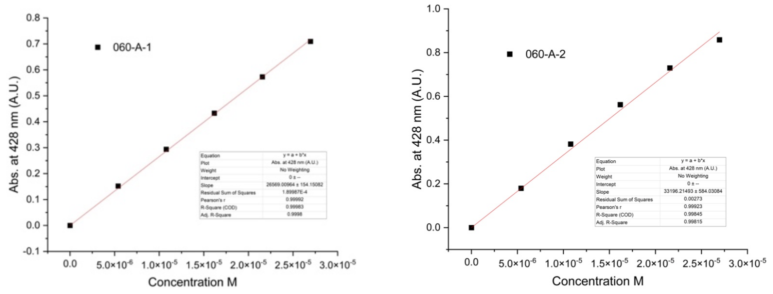

Figure S17. Graphical plots used (in triplicate) for the molar absorption coefficient calculation of [Ir(**L4**)$_2$(en)]Cl in mixtures of buffer (25 mM MOPS and 50 mM sodium chloride at pH 7) and DMSO.

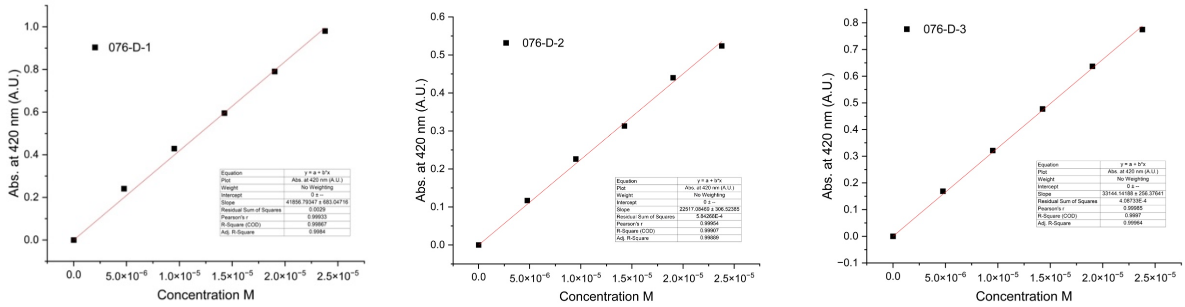

Figure S18. Graphical plots used (in triplicate) for the molar absorption coefficient calculation of [Ir(**L5**)$_2$(en)]Cl in mixtures of buffer (25 mM MOPS and 50 mM sodium chloride at pH 7) and DMSO.

Table S4. Calculated molar absorption coefficients for the longest wavelength absorption band of the complexes in mixtures of buffer (25 mM MOPS and 50 mM sodium chloride at pH 7) and DMSO.

	**[Ir(L1)$_2$(en)]Cl 436 nm**	**[Ir(L2)$_2$(en)]Cl 436 nm**	**[Ir(L3)$_2$(en)]Cl 428 nm**	**[Ir(L4)$_2$(en)]Cl 428 nm**	**[Ir(L5)$_2$(en)]Cl 420 nm**
ε / M^{-1} cm^{-1} (n=1)	28344.00673	27793.31167	37058.6476	26569.00964	41856.79347
ε / M^{-1} cm^{-1} (n=2)	26708.90419	32120.37409	30461.66889	33196.21493	22517.08469
ε / M^{-1} cm^{-1} (n=3)	19216.62687	26924.5531	37166.30898	27815.04923	33144.14188
ε / M^{-1} cm^{-1}	(24 ± 5) x 10^3	(28 ± 3) x 10^3	(34 ± 4) x 10^3	(29 ± 4) x 10^3	(32 ± 10) x 10^3

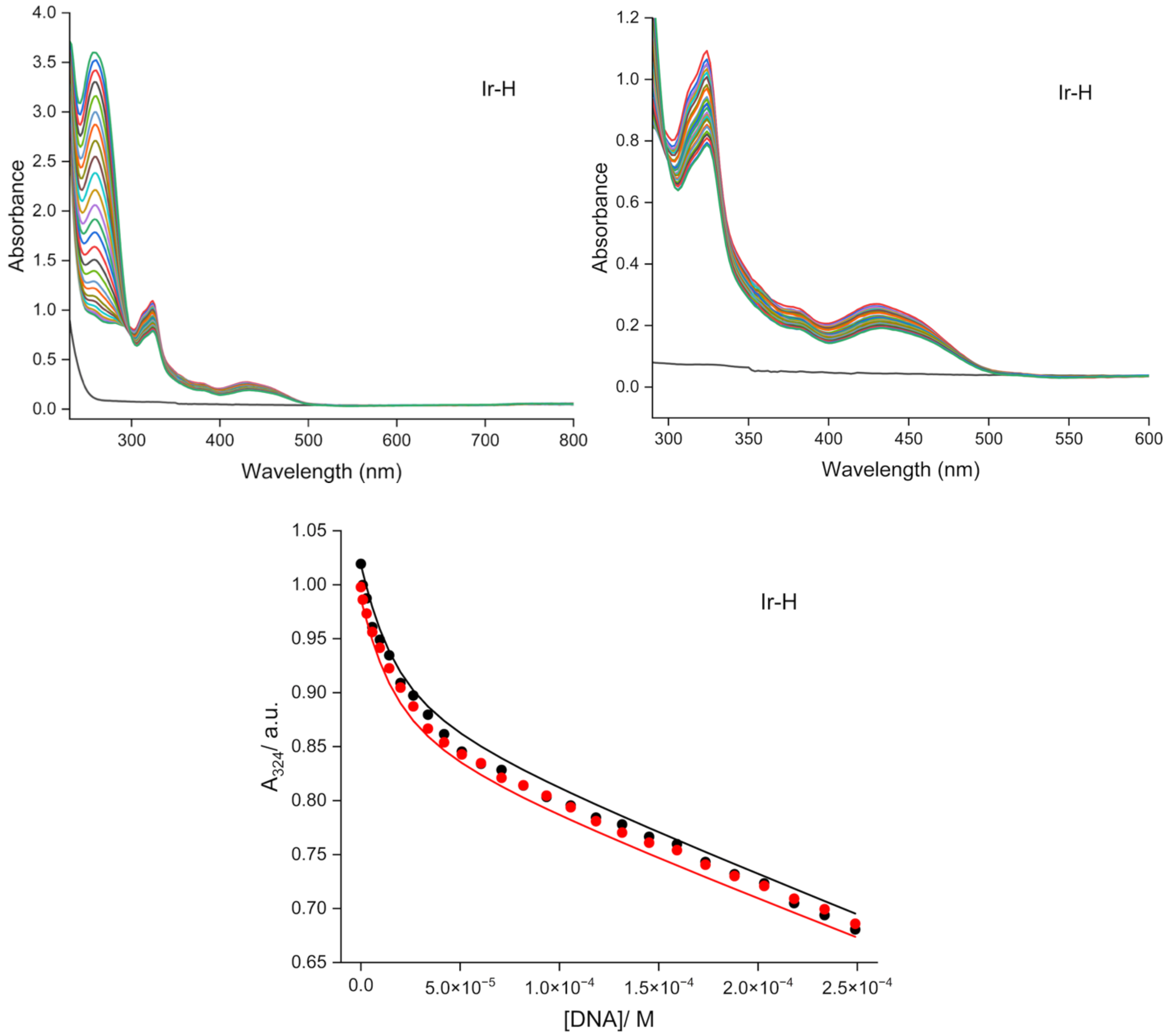

Figure S19. Fitted UV-vis titration data for [Ir(**L1**)$_2$(en)]Cl (44.14 μM) with sequential aliquots of FSDNA (25 mM MOPS, 5 mM NaCl, pH 7.00, at 25 °C).

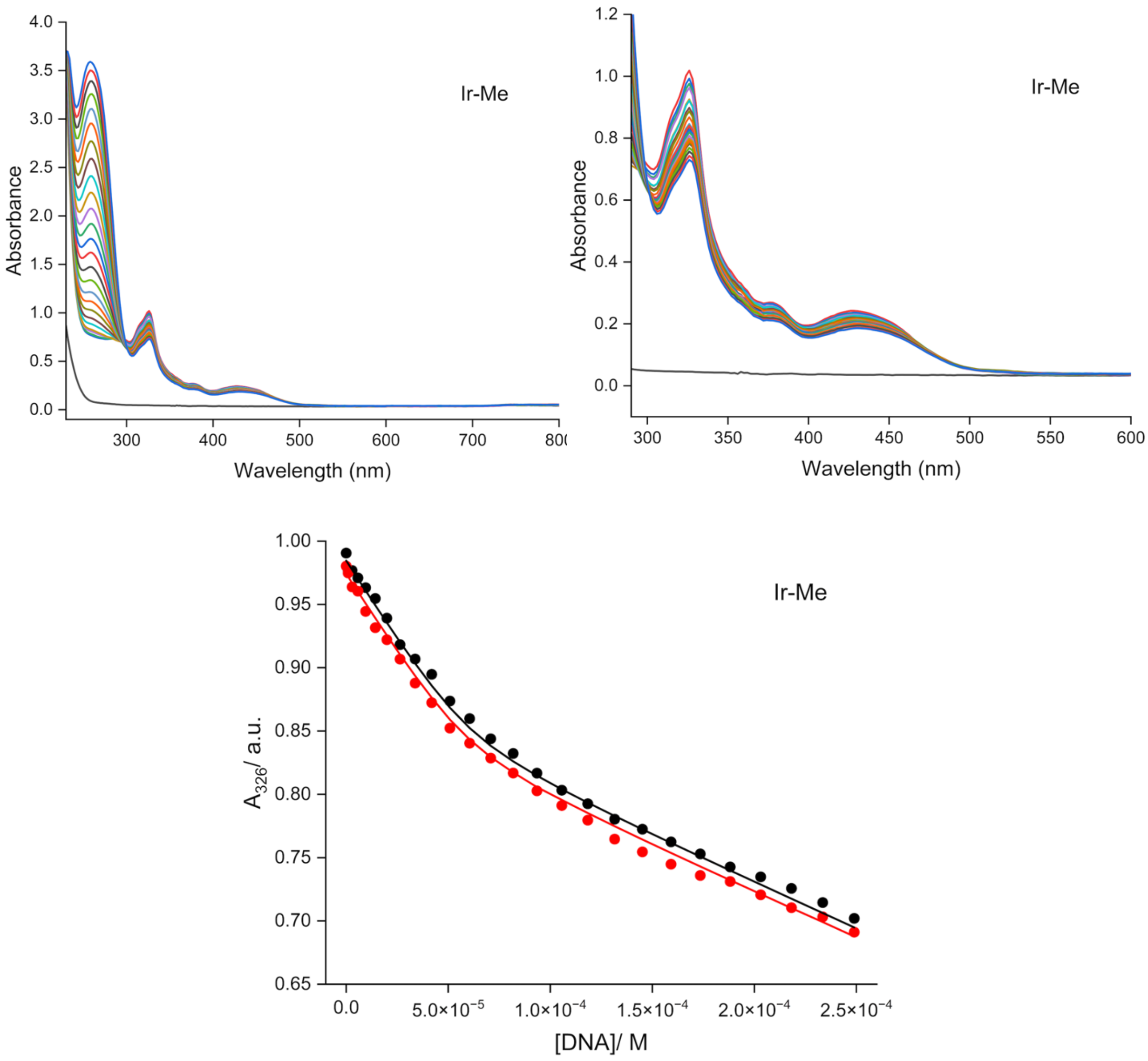

Figure S20. Fitted UV-vis titration data for [Ir(**L2**)$_2$(en)]Cl (35.17 μM) with sequential aliquots of FSDNA (25 mM MOPS, 5 mM NaCl, pH 7.00, at 25 °C).

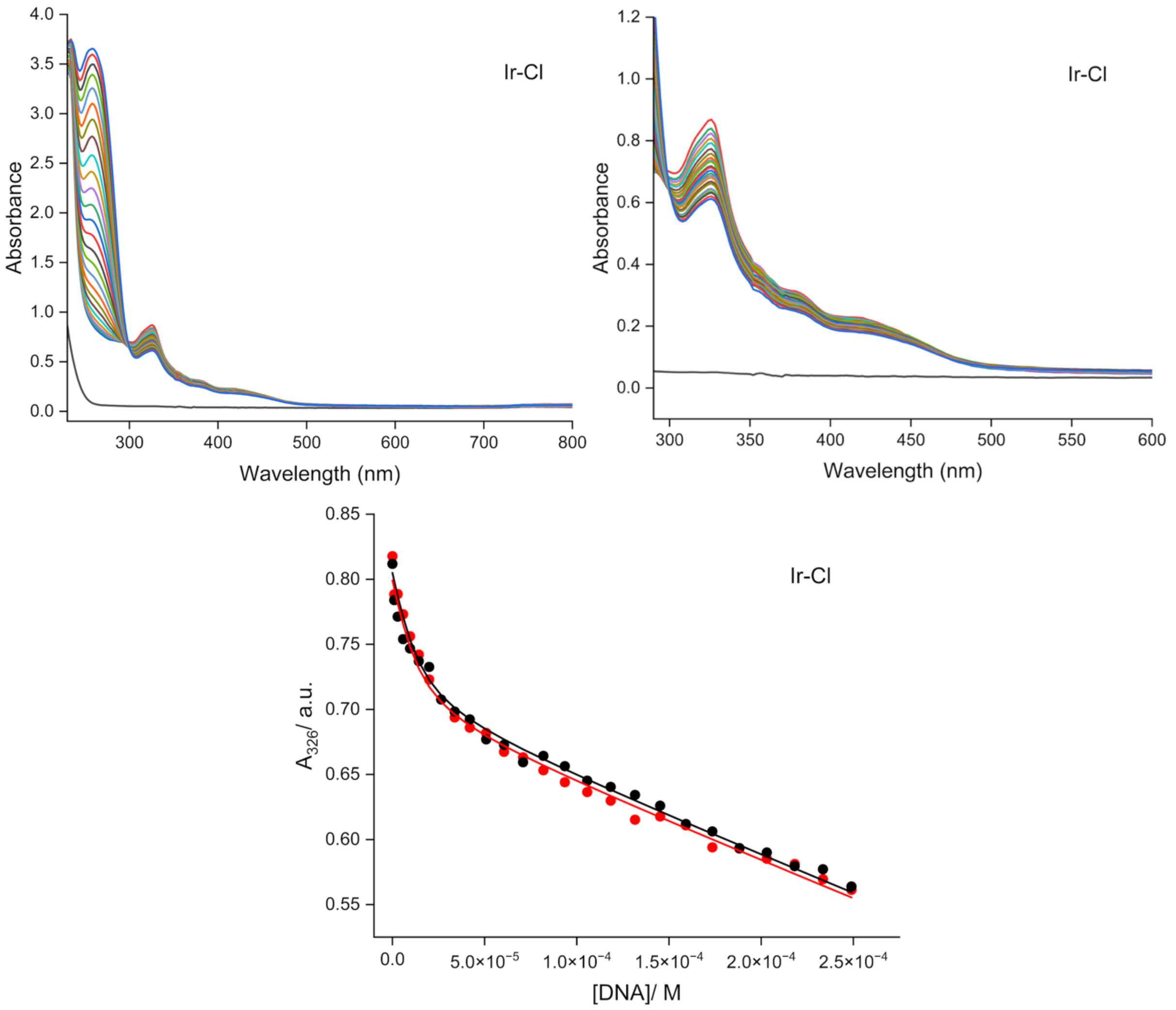

Figure S21. Fitted UV-vis titration data for [Ir(**L4**)$_2$(en)]Cl (29.62 μM) with sequential aliquots of FSDNA (25 mM MOPS, 5 mM NaCl, pH 7.00, at 25 °C).

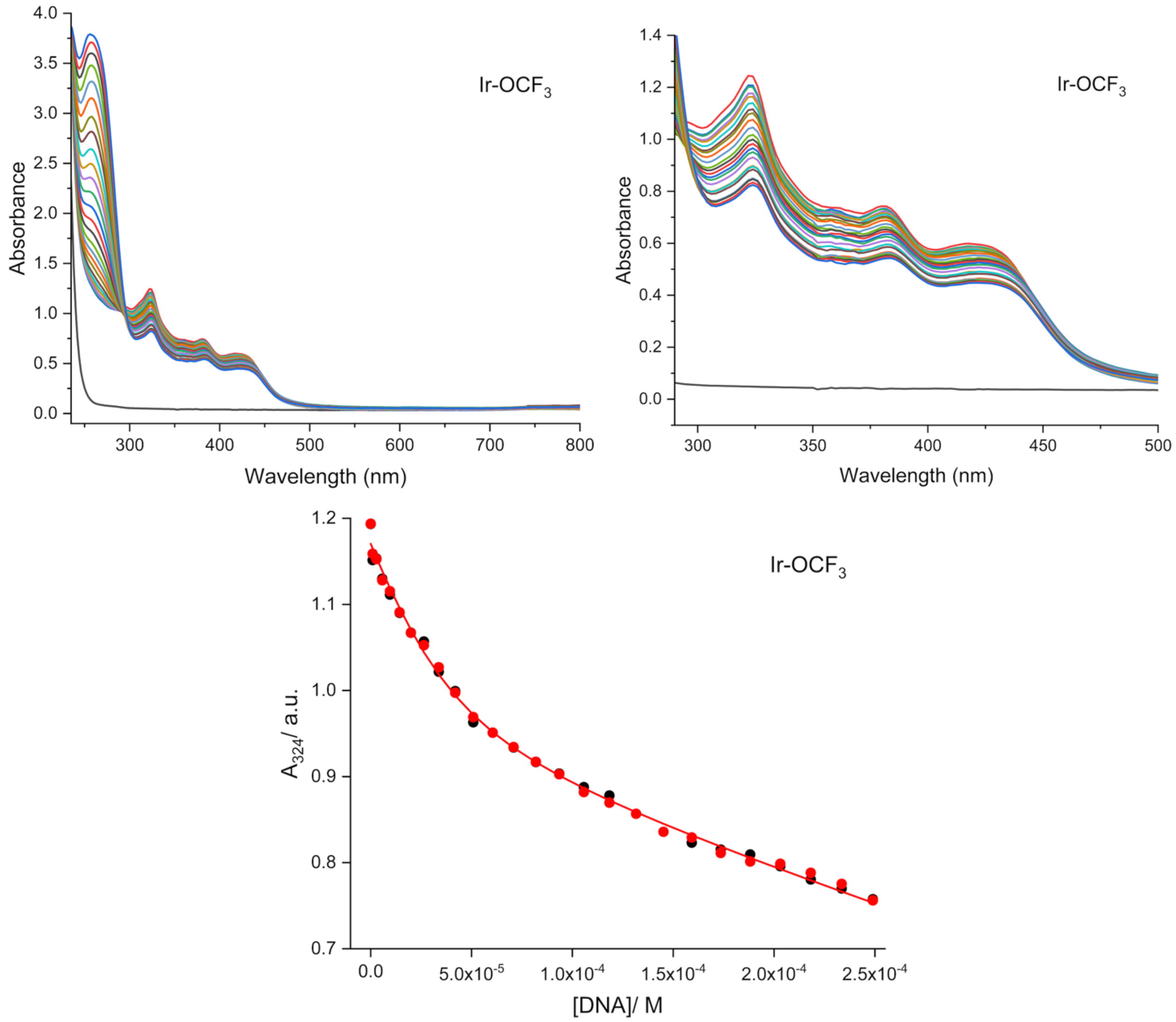

Figure S22. Fitted UV-vis titration data for [Ir(**L5**)$_2$(en)]Cl (37.55 μM) with sequential aliquots of FSDNA (25 mM MOPS, 5 mM NaCl, pH 7.00, at 25 °C).

Table S5. Sample conditions for the isothermal calorimetric measurements.

complex	focus on first binding event	focus on second binding event
**[Ir(L1)$_2$(en)]Cl**	300 and 244 µM complex into 200 µM DNA	978.2 µM complex into 200 µM DNA
**[Ir(L2)$_2$(en)]Cl**	327.1 µM complex into 350 µM DNA	327.1 µM complex into 45 µM DNA
**[Ir(L3)$_2$(en)]Cl**	184 µM complex into 200 µM DNA	739 µM complex into 200 µM DNA

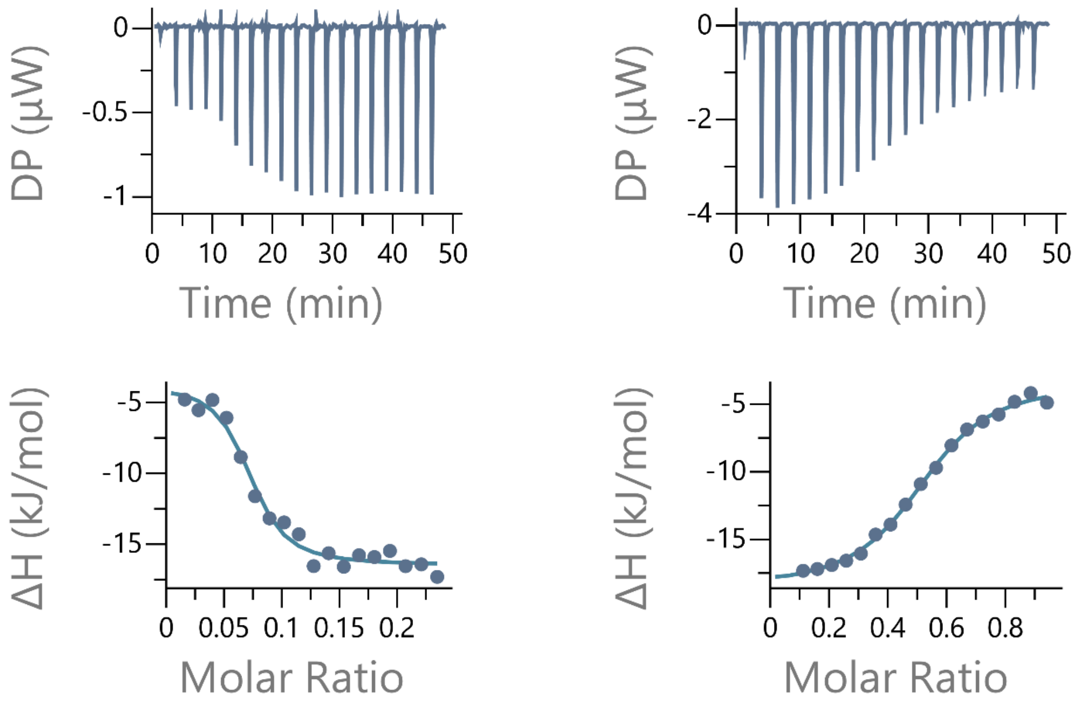

Figure S23. ITC data obtained for the addition of [Ir(**L1**)$_2$(en)]Cl to DNA.

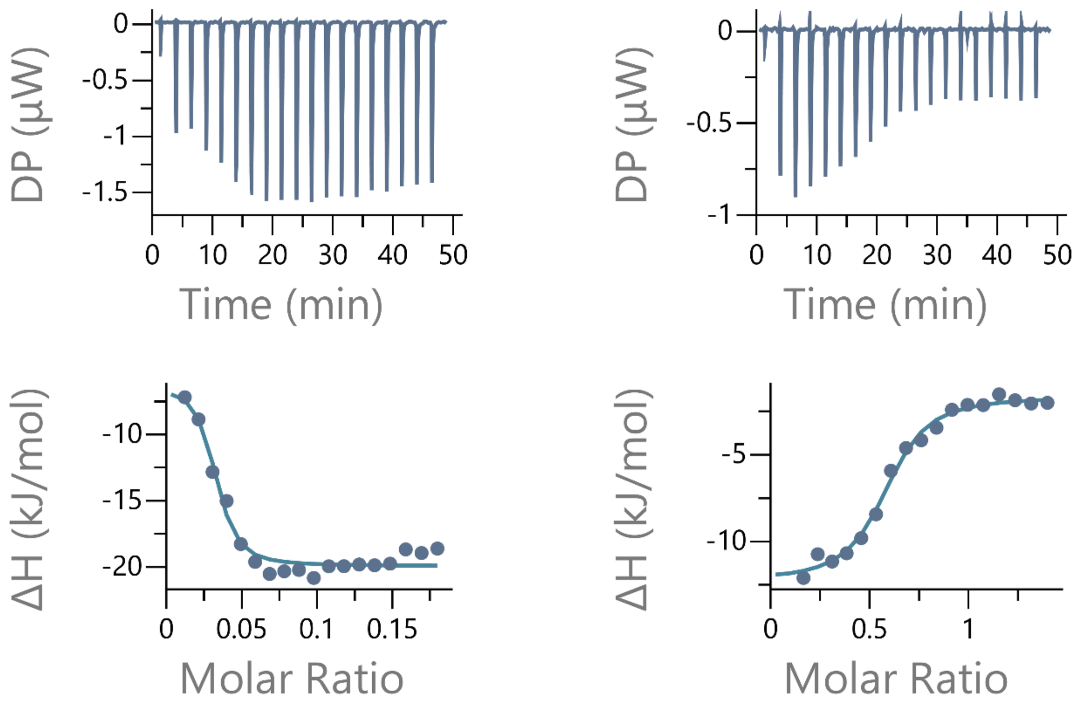

Figure S24. ITC data obtained for the addition of [Ir(**L2**)$_2$(en)]Cl to DNA.

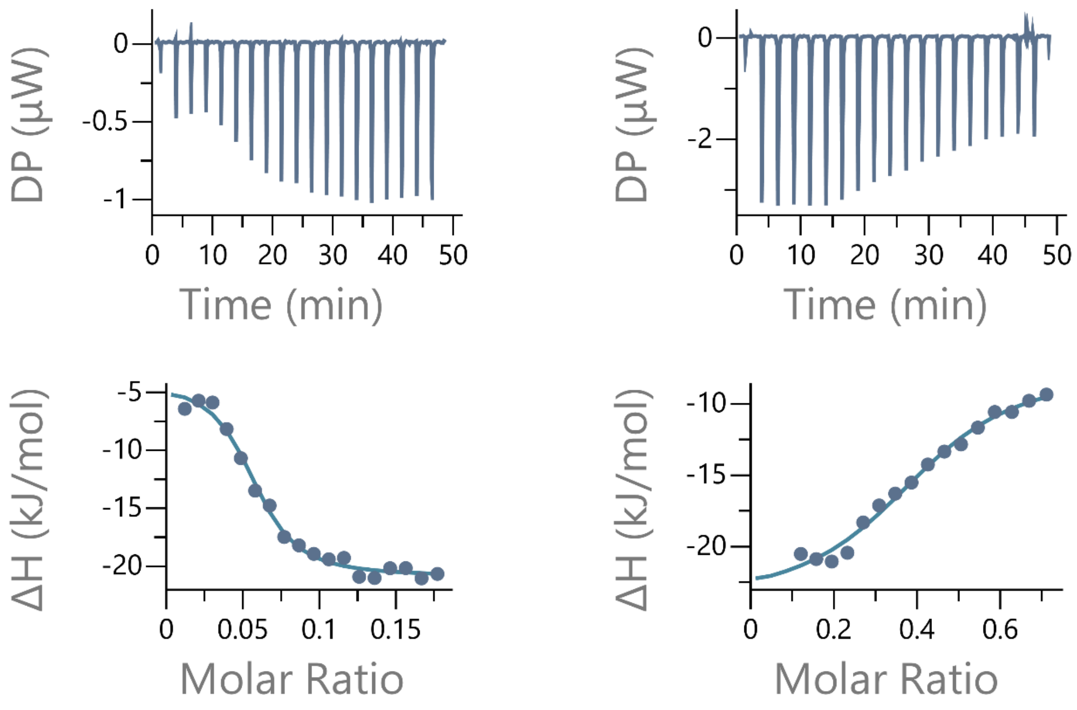

Figure S25. ITC data obtained for the addition of [Ir(**L3**)$_2$(en)]Cl to DNA.

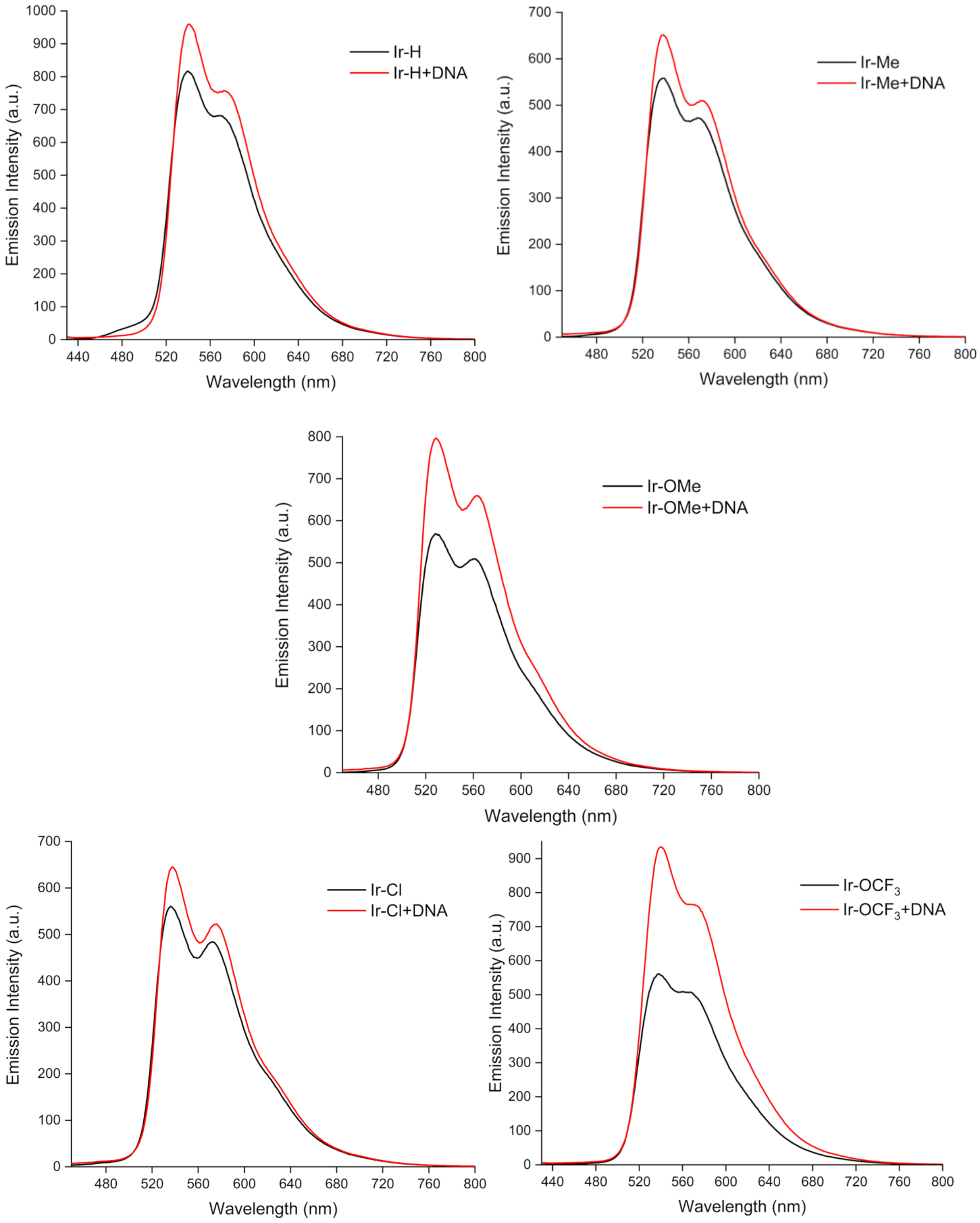

Figure S26. Photoluminescence steady state data before and after addition of FS DNA (recorded in MOPS and 5 mM NaCl with a pH of 7.00 at 298 K)

References

[1] K. Bahrami, M.M. Khodaei, F. Naali, *J. Org. Chem*. 2008, **73**, 6835
[2] M. Yoritate, A.T. Londregan, Y. Lian, J.F. Hartwig, *J. Org. Chem*. 2019, **84**, 15767
